# The Hippo pathway integrates PI3K–Akt signals with mechanical and polarity cues to control tissue growth

**DOI:** 10.1371/journal.pbio.3000509

**Published:** 2019-10-15

**Authors:** Nerea Borreguero-Muñoz, Georgina C. Fletcher, Mario Aguilar-Aragon, Ahmed Elbediwy, Zoé I. Vincent-Mistiaen, Barry J. Thompson

**Affiliations:** 1 Epithelial Biology Laboratory, The Francis Crick Institute, London, United Kingdom; 2 EMBL Australia, Department of Cancer Biology & Therapeutics, The John Curtin School of Medical Research, The Australian National University, Acton, Australia; Rutgers The State University of New Jersey, UNITED STATES

## Abstract

The Hippo signalling pathway restricts cell proliferation in animal tissues by inhibiting Yes-associated protein (YAP or YAP1) and Transcriptional Activator with a PDZ domain (TAZ or WW-domain–containing transcriptional activator [WWTR1]), coactivators of the Scalloped (Sd or TEAD) DNA-binding transcription factor. *Drosophila* has a single YAP/TAZ homolog named Yorkie (Yki) that is regulated by Hippo pathway signalling in response to epithelial polarity and tissue mechanics during development. Here, we show that Yki translocates to the nucleus to drive Sd-mediated cell proliferation in the ovarian follicle cell epithelium in response to mechanical stretching caused by the growth of the germline. Importantly, mechanically induced Yki nuclear localisation also requires nutritionally induced insulin/insulin-like growth factor 1 (IGF-1) signalling (IIS) via phosphatidyl inositol-3-kinase (PI3K), phosphoinositide-dependent kinase 1 (PDK1 or PDPK1), and protein kinase B (Akt or PKB) in the follicular epithelium. We find similar results in the developing *Drosophila* wing, where Yki becomes nuclear in the mechanically stretched cells of the wing pouch during larval feeding, which induces IIS, but translocates to the cytoplasm upon cessation of feeding in the third instar stage. Inactivating Akt prevents nuclear Yki localisation in the wing disc, while ectopic activation of the insulin receptor, PI3K, or Akt/PKB is sufficient to maintain nuclear Yki in mechanically stimulated cells of the wing pouch even after feeding ceases. Finally, IIS also promotes YAP nuclear localisation in response to mechanical cues in mammalian skin epithelia. Thus, the Hippo pathway has a physiological function as an integrator of epithelial cell polarity, tissue mechanics, and nutritional cues to control cell proliferation and tissue growth in both *Drosophila* and mammals.

## Abstract 2

This study shows that the PI3K-Akt pathway promotes cell proliferation via the Yorkie/YAP transcriptional coactivator in response to mechanical stretching in vivo; the Hippo kinase acts as a key integrator of Akt and mechanical cues to control Yorkie/YAP activity and cell proliferation, with implications for human cancer, in which PI3K-Akt and mechanical forces play key roles in driving cell proliferation and malignancy.

## Introduction

The growth of animal tissues is known to depend on nutritionally induced circulating hormones of the insulin/insulin-like growth factor 1 (IGF-1) family [1–3]. In addition, tissue growth can be mechanically induced via the Hippo signalling pathway [4–8]. How these two mechanisms of tissue growth control are integrated during animal development remains unclear. The fruit fly *Drosophila* offers a tractable model system to explore how insulin/IGF-1 signalling (IIS) and the Hippo pathway interact to influence cell proliferation in multiple epithelial tissues during development. In *Drosophila* oogenesis, the developing oocyte in each egg chamber is fed cytoplasm by endoreplicating germline nurse cells that grow enormously in size by cell growth without cell division [9–13]. The somatic follicle cell epithelium that surrounds the germline grows by coordinated cell growth and cell division—cell proliferation—to precisely encapsulate each egg chamber during early stages of oogenesis (stages 1–7) [14]. Proliferation of follicle cells is thought to occur in response to mechanical stretching caused by the growth of the underlying germline [15, 16]. Furthermore, growth of the entire egg chamber is known to depend on adequate nutrient intake by the adult female [17]. After fertilisation of the egg, embryogenesis proceeds within the eggshell without the need for feeding or growth. At the end of embryogenesis, the hatching larvae is able to feed and grow via a similar subdivision of tissues into those growing without cell division (the endoreplicating larval tissues) and those growing by cell proliferation (the mitotic ‘imaginal discs’ that give rise to the future adult body or ‘imago’) [11, 12, 18]. The growth rate of the entire larva, including both endoreplicating and proliferating tissues, once again depends upon nutritional intake and thus contributes to the determination of adult body size [1, 2, 19].

The IIS pathway was found to be induced by nutritional cues in *Drosophila* [1–3, 20–23]. Nutrient intake stimulates production of circulating *Drosophila* insulin-like peptides (Dilps), which activate the insulin/IGF-1 receptor (InR), leading to the recruitment of the phosphatidyl inositol-3-kinase (PI3K—composed of two subunits, p60 and p110), which coverts phosphatidyl inositol phosphate (PIP)2 to PIP3 at the plasma membrane of signal-receiving cells (a process antagonised by the phosphatase and tensin homolog PTEN) [3, 20–30]. Induction of PIP3 leads to the recruitment of two Pleckstrin Homology (PH)-domain–containing kinases, phosphoinositide-dependent protein kinase 1 (PDK1) and Akt (also called protein kinase B [PKB]), enabling PDK1 to phosphorylate Akt, which is then responsible for signal transduction [31–37] and tissue growth control [38–41].

One important downstream effector of Akt is the Target of Rapamycin (TOR) complex 1 (TORC1), which promotes cell growth by inducing translation via Ribosomal protein S6 kinase (S6K) [39, 42, 43] and inactivating the translational inhibitor, Elongation initiating factor 4E binding protein (4E-BP) [43, 44]. TORC1 also induces ribosome biogenesis via the RNA Polymerase (Pol) II transcription factors Myc [45–49] and DNA replication element binding factor (DREF) [50] and the RNA Pol I activators Transcription intermediary factor 1A (TIF1A/Rrn3) [51, 52] and TIF1B/SL1-Upstream binding factor (UBF) [53], as well as by inactivating the RNA Pol III inhibitor Maf1 [54, 55]. TORC1 is composed of the TOR kinase, Raptor, and Lst8 subunits [56–59] and is activated by Ras-homolog enriched in the brain (Rheb) GTPase [60–62] at the lysosome [63, 64] and inhibited by the Tuberous Sclerosis Complex TSC1/2 GTPase-activating proteins (GAPs) [65–67] and the 40-kDa proline-rich Akt substrate (PRAS40) [68–70]. Akt appears to activate TORC1 by phosphorylating TSC2 to displace it from the lysosome [71, 72], although mutation of two Akt phosphorylation sites in TSC2 does not affect growth in *Drosophila* [73], suggesting that other Akt targets, such as PRAS40, may compensate. Importantly, a negative feedback loop operates in which TORC1 activation promotes cell growth but also functions to reduce phosphorylation and activation of Akt itself [74, 75].

Another target of Akt is the transcription factor Forkhead box O (FOXO), which, upon IIS activation, becomes phosphorylated by Akt to induce its retention in the cytoplasm [76–78]. *Drosophila foxo* mutants are viable and normally sized, but *foxo* is required for complete suppression of tissue growth upon reduced insulin/IGF-1–PI3K–Akt signalling, which sends FOXO to the nucleus to inhibit cell proliferation and promote apoptosis [79, 80]. Thus, Akt can act independently of the TORC1 complex to control cell proliferation rather than cell growth, raising the question of whether additional Akt substrates may exist that contribute to the control of tissue growth by the IIS pathway in proliferating tissues. The importance of this question is underscored by the fact that activation of PI3K–Akt signalling (in *PI3K catalytic subunit alpha* [*PIK3CA*] constitutively active mutant or in *PTEN* inactivating mutant tumours) is a widespread cause of malignant human cancer, while activation of TORC1 (in *TSC1* or *TSC2* mutant tumours) causes benign hamartomas [75, 81].

The Hippo signalling pathway was discovered by *Drosophila* genetics to restrict the rate of cell proliferation and final tissue size in developing wing and eye imaginal discs but does not affect cell growth in endoreplicating tissues [4–6]. The upstream kinase Hippo (Hpo) activates the downstream kinase Warts (Wts), which then directly inhibits the nuclear localisation of Yorkie (Yki), a transcriptional coactivator for Scalloped (Sd)/TEA domain (TEAD) DNA-binding proteins [82–84]. In mutants for *hpo* or *wts*, Yki translocates to the nucleus to switch Sd/TEAD from a repressor to an activator, which then drives cell proliferation and tissue growth in the developing wing and eye imaginal discs [83–86]. When mutant clones for *yki* are induced in the wing disc, cell proliferation is inhibited, and cells are eliminated by apoptosis [82, 87]. These dramatic phenotypes strongly suggest that the Hippo pathway might have a physiological role in tissue growth control in proliferating tissues, but it is still unclear how dynamic regulation of Yki nuclear localisation is controlled as tissues initiate or arrest their proliferation during development.

A physiological role for the Hippo pathway as a sensor of mechanical stress and strain in vivo was recently identified in *Drosophila* [88, 89]. Here, we explore how mechanical regulation of Hippo pathway integrates with nutritional control of tissue growth via the IIS pathway in the ovarian follicle cell epithelium and wing imaginal disc. Previous work indicated that genetic alteration of IIS can influence Hippo pathway signalling, but the physiological relevance of this observation and the molecular mechanisms responsible are still not fully understood [90]. Here, we propose that insulin/IGF-1 act via Akt to inhibit Hippo pathway signalling and promote nuclear localisation of Yki in response to mechanical stress and strain. In the follicle cell epithelium, Yki responds primarily to mechanical strain driven by growth of the germline (itself insulin/IGF-1– and TORC1-dependent) but also requires PI3K–Akt signalling in follicle cells. In the wing imaginal disc, Yki responds to Fat–Dachsous signalling [4–8] and mechanical stress in columnar cells [89, 91–94], as well as to mechanical strain in flattened peripodial cells [88, 95], and we uncover an additional physiological regulation by nutritionally regulated IIS via PI3K–Akt. We further confirm a recent report that TORC1 is not required in the wing disc for nuclear localisation of Yki and that loss of TORC1 in fact drives Yki to the nucleus [96], which we attribute to feedback effects on PI3K–Akt. Finally, we confirm our findings in the mammalian skin epithelium, an example of a tissue in which both IIS and mechanically regulated Yes-associated protein (YAP) are crucial to promote cell proliferation.

## Results

**Fig 1:**
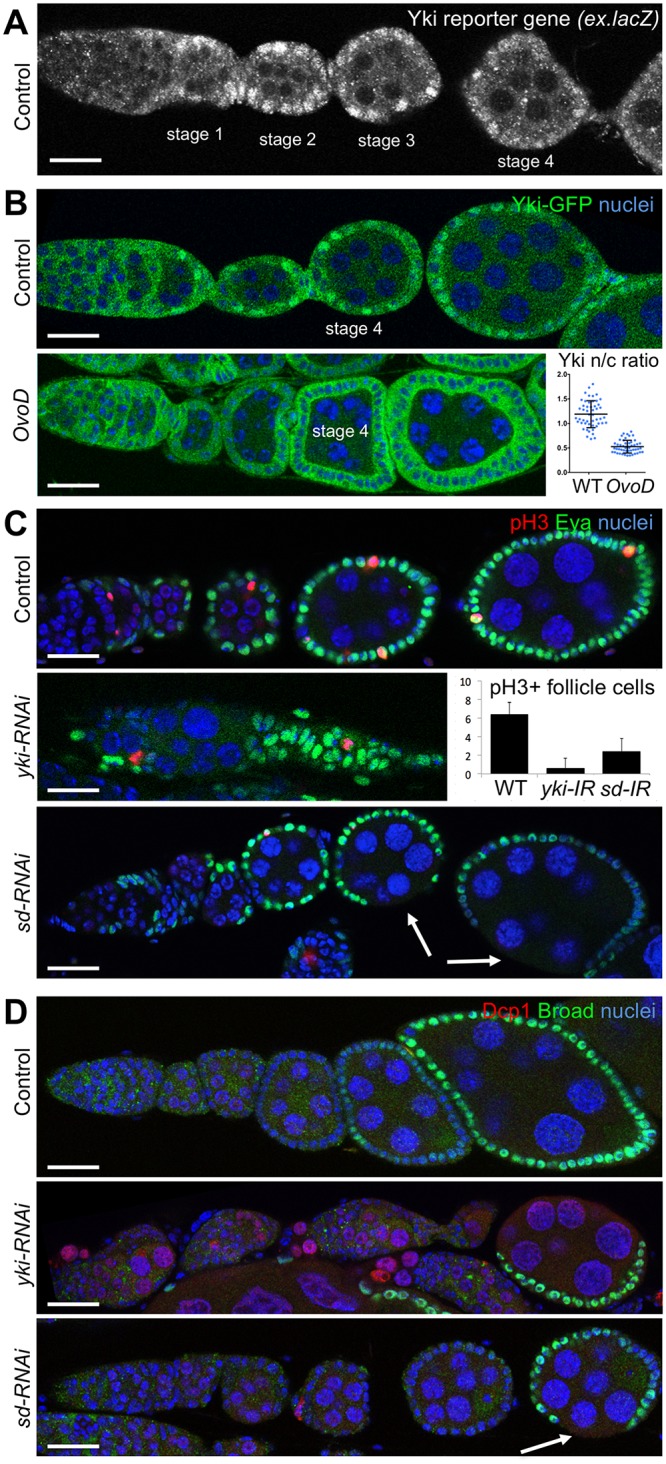
Yki localises to the nucleus to drive stretch-induced follicle cell proliferation in *Drosophila* egg chambers. A) Expression of the Yki–Sd reporter gene *ex*.*lacZ* in early egg chambers is revealed by the pattern of nuclear beta-galactosidase immunostaining. Note that *ex*.*lacZ* is expressed during the stages in which mechanical stretch drives follicle cell proliferation. B) Expression and subcellular localisation of a Yki–GFP knockin line in early egg chambers reveals nuclear localisation of endogenous Yki during the stages of stretch-induced follicle cell proliferation. Quantification of the nuclear/cytoplasmic ratio of Yki–GFP is shown on the right (*n* > 30 per genotype; *t* test *p* < 0.05). See supplementary file S1_Data.xlsx for underlying data. C) Mitotic cells (pH3+) in the follicle cell epithelium are reduced upon expression of Yki–RNAi or Sd–RNAi. Numbers of follicle cells are reduced by both Yki–RNAi or Sd–RNAi, more weakly in the latter case. The Yki–RNAi phenotype is so strong that germ line cells are often affected by insufficiency in follicle cell numbers. Follicle cell fate, marked by expression of Eya, is not affected. The graph shows quantification of the average number of mitotic cells (pH3+) per egg chamber in each of the genotypes above (*n* > 10 egg chambers per genotype). For the Yki–RNA quantification, weaker phenotypes that still form individual egg chambers were used to measure pH3+ cells per egg chamber. See supplementary file S1_Data.xlsx for underlying data. D). Apoptosis, marked by Dcp1-positive cells, does not occur in follicle cell epithelia expressing Yki–RNAi or Sd–RNAi but does occur in germline cells affected by insufficiency in follicle cell numbers upon expression of Yki–RNAi. Follicle cell fate, marked by expression of Broad at stage 6 onward, is not affected by either Yki–RNAi or Sd–RNAi. Scale bars approximately 10 μM. Dcp1, *Drosophila* Death Caspase 1; *ex*.*lacZ*, *expanded*.*lacZ*; Eya, Eyes Absent; GFP, green fluorescent protein; *IR*, Inverted Repeat Hairpin RNAi; n/c, nuclear/cytoplasmic ratio; *OvoD*, Ovo dominant; pH3+, phospho-Histone-H3–positive; RNAi, RNA interference; Sd, Scalloped; WT, wild type; Yki, Yorkie.

We first examined early *Drosophila* oogenesis, where growth of the germline was shown to mechanically induce proliferation of surrounding follicle cells during stages 2–6 [15, 16]. We find that expression of the Yki reporter gene *expanded*.*lacZ* (*ex*.*lacZ*) is elevated and that green fluorescent protein (GFP)-tagged Yki localises to the nucleus in proliferating follicle cells of early-stage egg chambers (Fig 1A and 1B and S1 Fig). Since *ex*.*lacZ* suffers from perdurance of the *lacZ*-encoded beta-galactosidase protein, which can obscure effects over short timescales, we chose to focus on Yki–GFP localisation as a more dynamic readout. We noticed that Yki nuclear localisation exhibited some female-to-female variation, evident in the quantification of the nuclear/cytoplasmic ratio of wild-type females (Fig 1B). We wondered whether this variation might relate to the growth rate of the egg chambers. To test this notion, we arrested germline growth beyond stage 4 using Ovo dominant (*OvoD*) mutants, which we find prevents mechanical stretching of follicle cells and inhibits Yki nuclear localisation (Fig 1B). Note that in *OvoD* egg chambers, some nuclear Yki is still detectable prior to stage 4, but after stage 4, Yki is strongly cytoplasmic, as shown in the quantification (Fig 1B). Yki is known to promote follicle stem cell maintenance [97] and polar cell fate [98], but whether Yki is also involved in stretch-induced cell proliferation in follicle cells has not been explored. We find that Yki is essential for normal follicle cell proliferation because silencing of *yki* expression specifically in follicle cells by *trafficjam*.*Gal4* (*tj*.*Gal4*)-driven transgenic Upstream activator sequence (*UAS*).*yki*-RNA interference (*RNAi*) reduces the number of phospho-Histone-H3–positive mitotic cells in each ovariole and profoundly disrupts oogenesis without affecting cell fate specification of follicle cells (marked by expression of Eyes Absent [Eya] and Broad) or follicle cell apoptosis (marked by *Drosophila* Death Caspase 1 [Dcp1] staining) (Fig 1C and 1D). Note that the insufficient number of follicle cells in the absence of Yki leads to germline apoptosis (Dcp1-positive) [16] (Fig 1D). Silencing of Sd by expression of *UAS*.*sd-RNAi* causes a milder phenotype, reducing follicle cell proliferation without disrupting germline growth during early stages (Fig 1C and 1D and S2 Fig). These results show that Yki subcellular localisation is dynamically patterned during early proliferative follicle cell development, becoming nuclear-localised in order for the Yki–Sd transcription factor to promote follicle cell proliferation during early egg chamber growth.

The fact that the loss of Sd causes a weaker phenotype than loss of Yki is expected because Sd acts as default repressor such that loss of Yki prevents activation and leads to constitutive repression of Yki–Sd target genes, while loss of Sd prevents either activation or repression of target genes [86, 99]. Nevertheless, we sought to confirm that our *UAS*.*sd-RNAi* line was indeed causing a full loss of Sd function. Since Sd acts as a direct binding partner for Yki in the nucleus, it promotes Yki nuclear localisation [100–102]. We find that expression of *UAS*.*sd-RNAi* is sufficient to completely abolish Yki nuclear localisation in the follicle cell epithelium of both early- and late-stage egg chambers (S3A and S3B Fig). We therefore characterised the *UAS*.*sd-RNAi* phenotype in more detail and found that the resulting adult female animals are completely sterile because of arrest of egg chamber development at stage 10 of oogenesis (S3B Fig). When we examine such arrested stage 10 egg chambers, we observe that they lack sufficient follicle cells to cover the surface of the egg chamber, leading to subsequent apoptosis of germline cells (Dcp1-positive) (S3C Fig). These results show that strong Sd loss of function disrupts follicle cell proliferation such that there are insufficient cells to cover the germline and ensure its survival beyond stage 10 of oogenesis. This Sd loss-of-function phenotype confirms a physiological requirement for regulation of the Hippo pathway during follicle cell proliferation in vivo.

**Fig 2:**
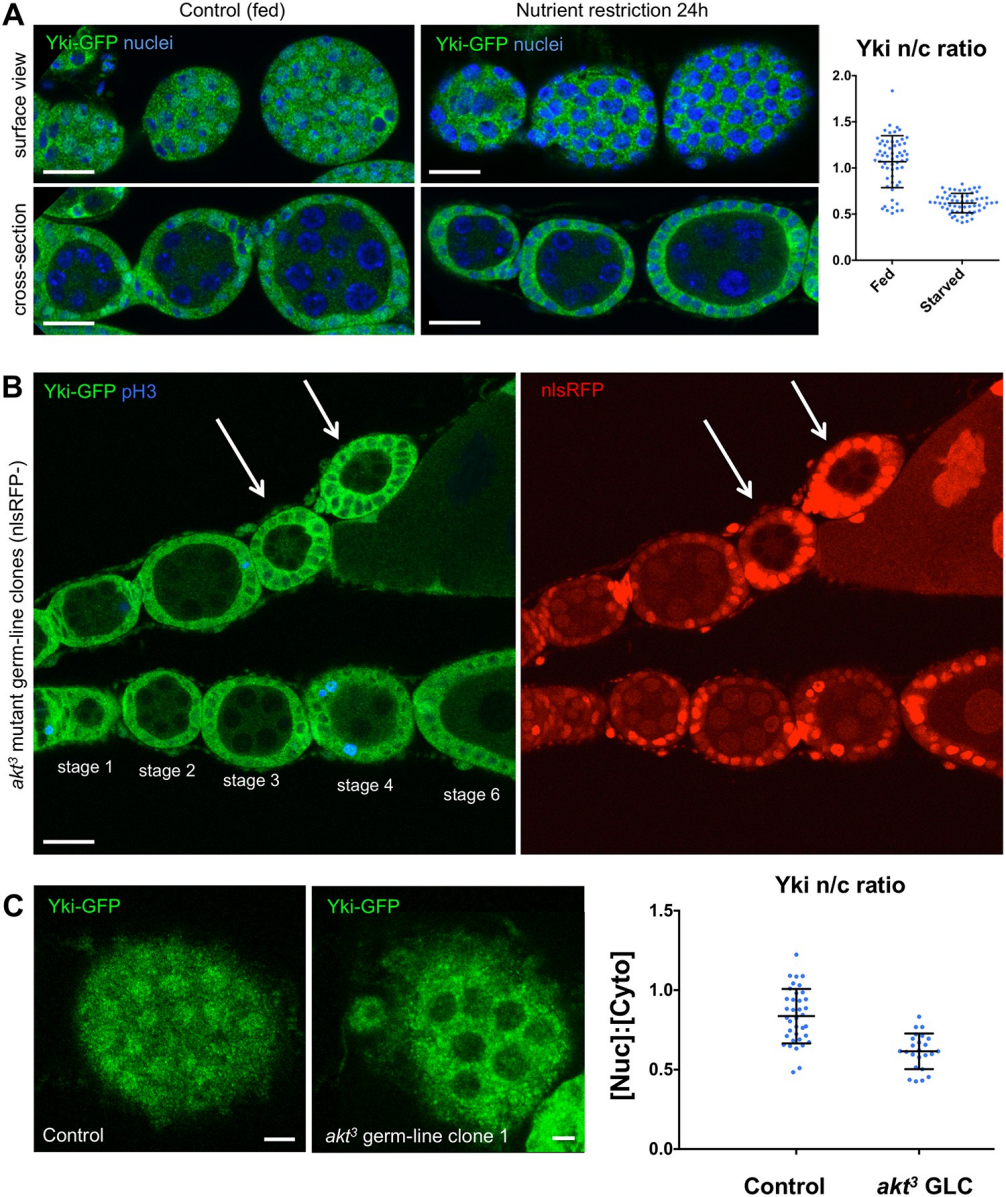
Insulin–PI3K–Akt signalling stimulates germline cell growth to drive mechanical stretching of follicle cells and Yki–GFP nuclear localisation. A) Yki–GFP localises to the nucleus in early-stage ovarian follicle cells but relocalises to the cytoplasm upon nutrient restriction of adult females for 24 h. Quantification of the nuclear/cytoplasmic ratio is shown on the right (*n* > 30 per genotype, *t* test *p* < 0.05). Scale bars approximately 10 μM. See supplementary file S1_Data.xlsx for underlying data. B) Yki–GFP localises to the nucleus in early-stage ovarian follicle cells but relocalises to the cytoplasm upon induction of *akt*^*3*^ mutant clones in the germline, marked by the absence of nlsRFP. Note that follicle cells are wild type. Scale bars approximately 10 μM. C) Surface views of *akt*^*3*^ mutant germline clones from (B) allow quantification of n/c. Scale bars approximately 3 μM. See supplementary file S1_Data.xlsx for underlying data. GFP, green fluorescent protein; nlsRFP, nuclear red fluorescent protein; n/c, nuclear/cytoplasmic ratio; pH3, phospho-Histone H3; PI3K, phosphatidyl inositol-3-kinase; Yki, Yorkie.

Since both follicle cell proliferation and germline growth are known to depend profoundly on nutritionally induced IIS [16, 17, 103, 104], we tested whether nutritional regulation of IIS in the germline cells might affect Yki subcellular localisation in the overlying follicle cell epithelium. We first compared the localisation of Yki–GFP in ovarioles from control (fed) adult females with those subjected to nutrient restriction for 24 h. We found that nutrient restriction caused a dramatic reduction in the nuclear localisation of Yki–GFP in follicle cells (Fig 2A). To test whether the effects of nutrient restriction were mediated by IIS to the germline, we induced null mutant clones of *akt*^*3*^ in the germline. We found that, as expected, cell growth is dramatically impaired in *akt*^*3*^ mutant germline cells and that this leads to relocalisation of Yki–GFP to the cytoplasm in the overlying follicle cell epithelium, which exhibits reduced cell proliferation (Fig 2B). The effect of Akt on germline growth is mediated by TORC1 because expression of *UAS*.*TOR-RNAi* in germline cells with the maternal *tubulin*.*Gal4* driver reduces germline cell growth and is sufficient to reduce mechanical stretching and nuclear localisation of Yki–GFP in the surrounding follicle cell epithelium (S4 Fig). Thus, IIS acts on the germline to drive cell growth and thus indirectly induces mechanical stretching of the follicular epithelium and nuclear localisation of Yki in follicle cells.

**Fig 3:**
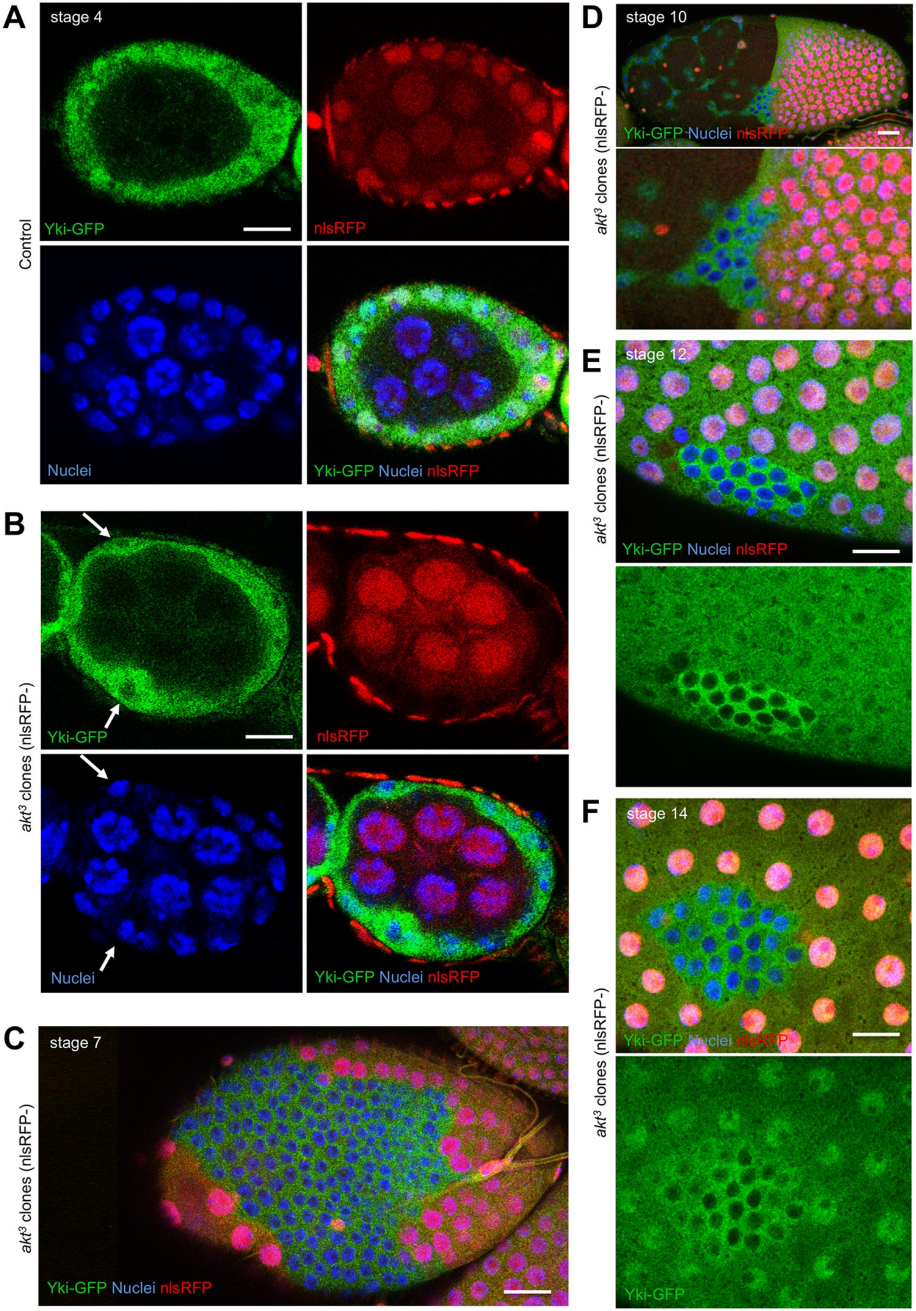
Insulin–PI3K–Akt signalling is required for nuclear localisation of Yki–GFP in the follicle cell epithelium. A) Yki–GFP localisation to the nucleus is detectable in follicle cells at stage 4 of oogenesis. Scale bars approximately 10 μM. B) Yki–GFP localisation to the nucleus is weak or absent in follicle cells at stage 4 of oogenesis that are mutant for *akt*^*3*^. Note that germline cells are wild type. Scale bars approximately 10 μM. C) Yki–GFP localisation to the nucleus is weak or absent in follicle cells at stage 7 of oogenesis that are mutant for *akt*^*3*^. Scale bars approximately 10 μM. D) Yki–GFP localisation to the nucleus is weak or absent in follicle cells at stage 10 of oogenesis that are mutant for *akt*^*3*^. Scale bars approximately 10 μM. E) Yki–GFP localisation to the nucleus is weak or absent in follicle cells at stage 12 of oogenesis that are mutant for *akt*^*3*^. Scale bars approximately 10 μM. F) Yki–GFP localisation to the nucleus is weak or absent in follicle cells at stage 14 of oogenesis that are mutant for *akt*^*3*^. Scale bars approximately 10 μM. GFP, green fluorescent protein; nlsRFP, nuclear red fluorescent protein; Yki, Yorkie.

We next tested whether IIS in the follicle cell epithelium itself affects Yki subcellular localisation because this pathway has previously been shown to act via PI3K and PDK1 to promote Yki activity in imaginal discs [90]. We sought to examine the role of the key downstream effector of these kinases, Akt, whose potential role was previously overlooked because RNAi of Akt in S2 cells did not completely abolish the effect of insulin on Yki phosphorylation [90]. We therefore induced mutant clones of *akt*^*3*^ in the follicle cell epithelium and found that Yki–GFP relocalises to the cytoplasm in these clones, even following strong cellular stretching at late stages of oogenesis (Fig 3A–3F).

**Fig 4:**
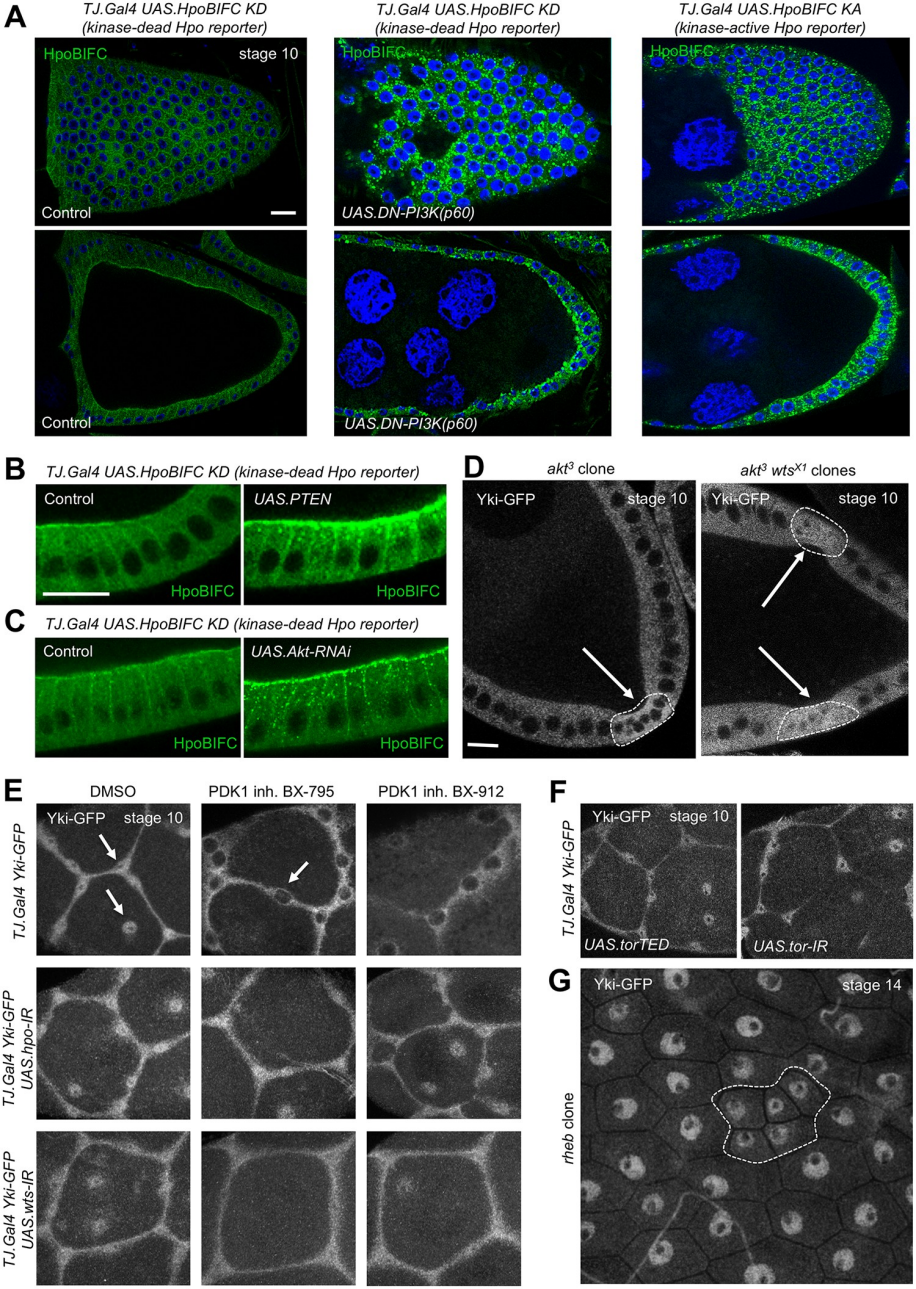
Insulin–PI3K–PDK1–Akt signalling to the follicle cell epithelium inhibits Hpo to drive nuclear Yki–GFP. A) Expression of an Hpo KD BIFC sensor is detectable at the apical membrane of columnar follicle cells at stage 10 of oogenesis and is dramatically up-regulated upon coexpression of a dominant-negative PI3K p60 subunit (UAS.p60). A KA Hpo BIFC sensor is also dramatically up-regulated compared to the KD version. Scale bars approximately 10 μM. B) Expression of PTEN up-regulates the KD Hpo BIFC sensor in columnar follicle cells at stage 10 of oogenesis. Scale bars approximately 10 μM. C) Expression of Akt–RNAi up-regulates the KD Hpo BIFC sensor in columnar follicle cells at stage 10 of oogenesis. D) Yki–GFP localisation to the nucleus is weak or absent in follicle cells at stage 10 of oogenesis that are mutant for *akt3* but is nuclear-localised in *akt*^*3*^ *wts*^*X1*^ double mutant clones. Scale bars approximately 10 μM. E) Yki–GFP localises to the nucleus in stretch cells at stage 10 of oogenesis. Inhibiting PDK1 activity acutely with BX-795 or BX-912 blocks Yki–GFP nuclear localisation in a manner that depends on the canonical Hippo pathway kinases Hpo and Wts, whose knockdown with RNAi restores Yki–GFP nuclear localisation even in the presence of PDK1 inhibitors. F) Yki–GFP localises to the nucleus even upon inhibition of TORC1 by expression of dominant-negative TOR-TED or TOR-RNAi in stretch cells at stage 10 of oogenesis. G) Yki–GFP localises to the nucleus even upon inhibition of TORC1 by induction of *rheb* mutant clones at stage 14 of oogenesis. BIFC, bimolecular fluorescence complementation; *DN*, dominant-negative; GFP, green fluorescent protein; Hpo, Hippo; inh., inhibited; *IR*, Inverted Repeat Hairpain RNAi; KA, kinase-active; KD, kinase-dead; PDK1, phosphoinositide-dependent protein kinase 1; PI3K, phosphatidyl inositol-3-kinase; PTEN, phosphatase and tensin homolog; Rheb, Ras-homolog enriched in the brain; RNAi, RNA interference; *tj*.*Gal4*, *trafficjam*.*Gal4*; TOR, Target of Rapamycin; TORC1, TOR complex 1; *UAS*, Upstream activator sequence; Wts, Warts; Yki, Yorkie.

PI3K–PDK1–Akt signalling acts by regulating the canonical Hippo pathway because an Hpo dimerisation reporter, normally detectable only in columnar follicle cells and not in stretched cells [88], is strongly up-regulated upon inhibition of PI3K or Akt or overexpression of PTEN (Fig 4A–4C). The effect of Akt depends on canonical Hippo pathway signalling because double mutant *akt*^*3*^ *wts*^*X1*^ clones still exhibit nuclear Yki–GFP localisation, even in columnar cells (Fig 4D). Furthermore, Yki nuclear localisation in highly stretched cells can be inhibited by treatment with two different PDK1 inhibitors, BX-795 and BX-912, whose effects are blocked by coexpression of *UAS*.*Hpo-RNAi* or *UAS*.*Wts-RNAi* (Fig 4E). The effect of Akt in follicle cells is not mediated by TORC1 because expression of dominant-negative *UAS*.*TOR*^*TED*^ or *UAS*.*TOR-RNAi* or induction of mutant clones for *rheb* does not prevent nuclear localisation of Yki–GFP (Fig 4F and 4G). Together, the above results show that IIS, via PI3K–PDK1–Akt, directly regulates the canonical Hippo pathway and is a prerequisite for nuclear localisation of Yki in both proliferating early-stage follicle cells and flattening late-stage stretch cells.

**Fig 5:**
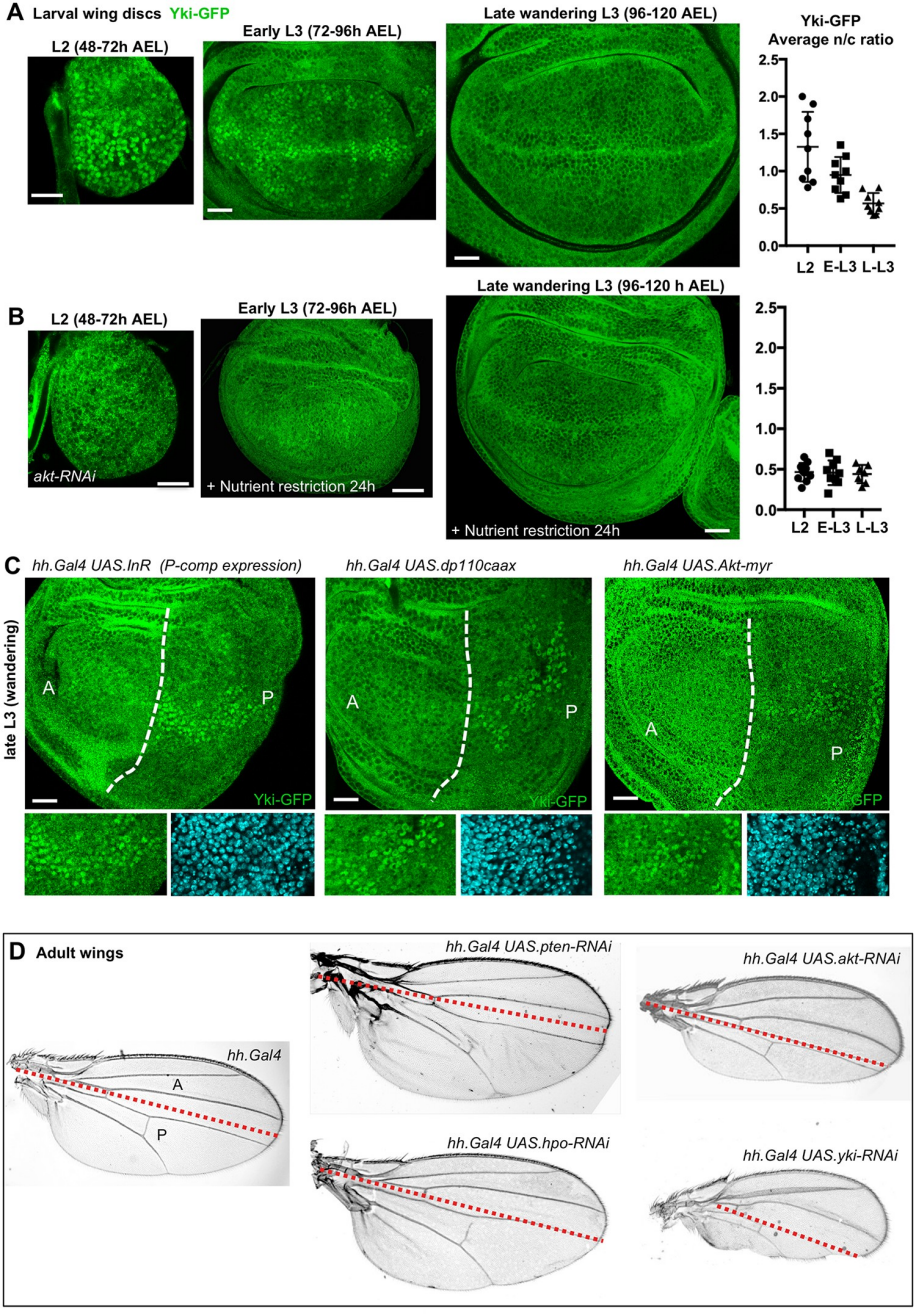
Yki becomes nuclear in response to both mechanical inputs and nutritionally induced insulin/IGF-1 signals in the developing wing. A) Yki–GFP localises to the nucleus in early second and third instar larval wing imaginal discs in a pattern that closely resembles that of mechanical stress and strain (see also S7 Fig). Yki–GFP localises much less strongly to the nucleus in late ‘wandering’ third instar larval wing imaginal discs. Note the cytoplasmic localisation in the central region and weak nuclear localisation at the dorsal ventral boundary and at the periphery at late L3. Quantification is shown on right (*n* > 10 per time point). Scale bars approximately 5 μM. See supplementary file S1_Data.xlsx for underlying data. B) Silencing of *akt* expression by *UAS*.*akt-RNAi* hairpin transgene prevents nuclear Yki–GFP localisation in the L2 wing disc. Reducing Akt signalling by nutrient restriction for 24 h also prevents nuclear localisation of Yki–GFP at early L3. Quantification is shown on right (*n* > 10 per time point). Scale bars approximately 5 μM. See supplementary file S1_Data.xlsx for underlying data. C) Activating PI3K–Akt signalling by overexpression of insulin receptor (*hh*.*Gal4 UAS*.*InR*), a membrane-targeted version of the PI3K catalytic subunit (*hh*.*Gal4 UAS*.*dp110caax*), or membrane-targeted Akt (*hh*.*Gal4 UAS*.*akt-myr*) in the P compartment is sufficient to maintain the early pattern of Yki–GFP nuclear localisation in the posterior compartment of late third instar wing discs. Compare A and P compartments. Scale bars approximately 5 μM. D) Adult wings showing similar tissue overgrowth and undergrowth phenotypes in the posterior compartment when *hh*.*Gal4* is used to silence expression of *pten* or *hpo* (which causes P-compartment overgrowth) as well as *akt* and *yki* (which causes P-compartment undergrowth). A, anterior; AEL, after egg laying; *dp110caax*, *Drosophila* p110-caax; GFP, green fluorescent protein; *hh*.*Gal4*, *hedgehog*.*Gal4*; Hpo, Hippo; IGF-1, insulin-like growth factor 1; InR, insulin/IGF-1 receptor; n/c, nuclear/cytoplasmic ratio; P, posterior; PI3K, phosphatidyl inositol-3-kinase; PTEN, phosphatase and tensin homolog; RNAi, RNA interference; *UAS*, upstream activator sequence; Yki, Yorkie.

To confirm our findings in another *Drosophila* tissue, we examined the proliferating *Drosophila* larval wing imaginal disc, particularly the wing pouch, where the Hippo pathway has been shown to respond to gradients of Fat–Dachsous signalling [4–8] as well as physiological mechanical inputs, Ajuba–Wts-mediated stress sensing in the columnar cells of the wing pouch, and Hippo-pathway–mediated strain sensing in the overlying peripodial cells [89, 94] (S5–S7 Figs). Since experimental manipulation of IIS has been reported to influence Hippo pathway signalling via PI3K–PDK1 in the wing disc [90], we wondered whether physiological changes in IIS might affect Yki nuclear localisation during wing development. Towards the end of the third instar of larval development, feeding ceases, and larvae begin to wander out of the food as they prepare to form pupae and undergo metamorphosis. Wing disc cell proliferation slows and subsequently arrests at this point, with final size determined by a combination of reduced IIS from the brain and a surge of ecdysone signalling that triggers pupariation and metamorphosis [21, 23, 105]. We tested whether this physiological change in IIS might regulate Yki–GFP as the wing disc slows and arrests proliferation at the end of the third instar. We find that nuclear Yki–GFP localisation is prominent during early stages but is reduced by the late third instar stage when cessation of larval feeding occurs (Fig 5A and S7 Fig). Reducing Akt signalling by expression of *akt–RNAi* or subjecting early third instar larvae to nutrient restriction for 24 h was sufficient to reduce Yki–GFP nuclear localisation (Fig 5B). Furthermore, the pattern of Yki nuclear localisation in early third instar wing discs can be maintained in late third instar wing discs by expressing insulin receptor (*UAS*.*InR*) or constitutively active forms of PI3K (*UAS*.*dp110caax*) or Akt (*UAS*.*Akt-myr*) in the posterior compartment with the *hedgehog*.*Gal4* (*hh*.*Gal4*) driver (Fig 5C). We confirm that *hh*.*Gal4*-driven manipulation of PI3K–Akt signalling and manipulation of Yki activity in wing discs generates similar tissue growth phenotypes in adult wings, illustrating the importance of cell proliferation control in larval imaginal discs for determination of final tissue size (Fig 5D). These results support the notion that the interplay of Fat–Dachsous signalling and mechanical cues with nutritionally induced IIS, acting via PI3K–PDK1–Akt, controls Yki nuclear translocation to promote tissue growth in the *Drosophila* wing.

A recent report suggested that IIS also acts via TOR to regulate Yki [96]. Interestingly, inhibition of TOR caused a strong ectopic localisation of Yki to the nucleus, even in late third instar imaginal discs [96]. We confirmed these findings with our Yki–GFP line, which strongly accumulates in the nucleus when dominant-negative *UAS*.*TOR*^*TED*^ is expressed in the posterior compartment of the wing disc (S8A Fig). The increase in nuclear Yki–GFP is due to disruption of the TORC1–Akt negative feedback loop [74] because the effects of *UAS*.*TOR*^*TED*^ are reversed by treatment with PDK1 inhibitors (S8B Fig). Thus, IIS promotes cell proliferation in the wing disc via PI3K–PDK1–Akt inhibition of the Hippo pathway and additionally promotes cell growth via TORC1, which also provides negative-feedback–limit Akt signalling. Accordingly, ectopic activation of IIS drives both cell growth and division, producing larger tissues with larger cells [3, 106], while ectopic activation of Yki produces larger tissues composed of increased numbers of normally sized cells [82].

**Fig 6:**
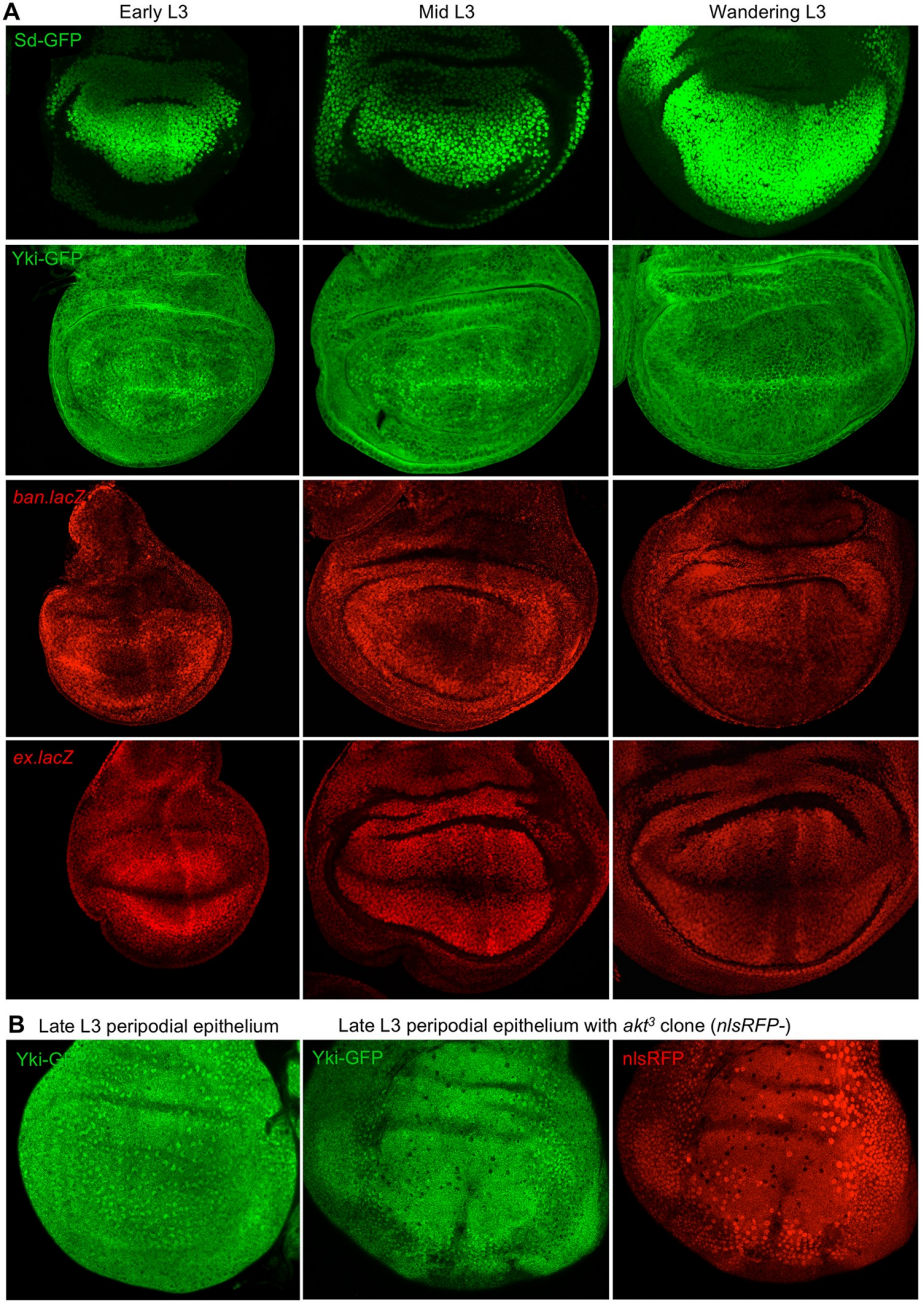
Patterns of Sd–GFP, Yki–GFP, and Yki target genes in the wing disc and loss of nuclear Yki–GFP in *akt*^*3*^ clones. A) Wing imaginal discs at early, mid, and late L3 showing expression patterns for Sd–GFP, Yki–GFP and two Yki target genes: *ban*.*lacZ* and *ex*.*lacZ*. Note the correlation between Yki–GFP nuclear localisation and its target genes at early L3, at which both are elevated in a circumferential pattern. Note also that the pattern of *lacZ* (beta-galactosidase) staining is maintained into late-stage L3 because of the perdurance of beta-galactosidase protein. Finally, both *ban*.*lacZ* and *ex*.*lacZ* target genes are normally repressed at the D–V boundary by Notch signalling, which prevents nuclear Yki–GFP from inducing them in this territory of the wing. Thus, only the circumferential activation of Yki–GFP is normally functional in the wing pouch, consistent with its proposed role in wing growth. B) Late L3 wing imaginal disc peripodial epithelial cells are strongly stretched and retain nuclear Yki–GFP. Note that this nuclear Yki–GFP localisation still depends on the low levels of Akt signalling occurring at this stage because the localisation becomes cytoplasmic in *akt*^*3*^ mutant clones (marked by the absence of nlsRFP). *ban*.*lacZ*, bantam-lacZ; D–V, Dorsal–Ventral; *ex*.*lacZ*, *expanded*.*lacZ*; GFP, green fluorescent protein; nlsRFP, nuclear red fluorescent protein; Sd, Scalloped; Yki, Yorkie.

We note that some differences exist between the spatiotemporal pattern of Yki–GFP localisation and the pattern of the Yki reporter genes *ex*.*lacZ* and *bantam*.*lacZ* (*ban*.*lacZ*) in the wing disc (Fig 6A). For example, the strong nuclear localisation of Yki–GFP at the Dorsal–Ventral (D–V) boundary is not reflected in either *ex*.*lacZ* or *ban*.*lacZ* expression, and both target genes are instead repressed at the D–V boundary (Fig 6A). Recent results explain this discrepancy because Notch signalling at the D–V boundary was found to potently repress the ability of activated Yki to transcriptionally induce target gene expression in the wing disc [107]. Thus, the combination of Fat–Dachsous signalling and mechanical and nutritional inputs determines Yki nuclear localisation in the wing, while other signalling pathways, such as Notch, can modulate the transcriptional output of Yki–Sd on specific target genes. Nevertheless, the resulting pattern of *ex*.*lacZ* or *ban*.*lacZ*—low medially and high in a circumferential pattern at the periphery (Fig 6A)—matches the pattern of Fat–Dachsous signalling and mechanical stretching previously proposed to complement the action of compartment boundary organisers [108, 109] and thus level out the rate of cell proliferation across the wing pouch [110–114].

To examine the mechanism by which Akt regulates the Hippo pathway in *Drosophila*, we tested whether a conserved Akt consensus phosphorylation site first identified in the human Hpo kinase homolog Mammalian Sterile 20 kinase (MST)2 in vitro [115–118] is required for control of tissue growth in *Drosophila*. The Akt phosphorylation site (T132) is located two amino acids downstream of the catalytic aspartate in the Hpo (and in human MST1/2) kinase domains, suggesting that Akt phosphorylation of might directly inhibit Hpo kinase activity (S9A–S9D Fig). A pan-Akt substrate phosphospecific antibody recognises the monomeric form of the Hpo kinase but not the active dimer, suggesting that phosphorylation by Akt may prevent dimerisation of Hpo (S9B Fig). We therefore tested whether mutation of the T132 residue would cause ectopic activation of the Hpo kinase in vivo. Using a landing site system for controlled expression of transgenes, we compared the effect of expressing wild-type Hpo versus phosphomutant HpoT132A in follicle cells. We find that wild-type Hpo mildly reduces the number of follicle cells, while HpoT132A dramatically reduces follicle cell numbers, exposing many gaps in the coverage of the germline (S9E Fig). Accordingly, Yki–GFP nuclear localisation is strongly inhibited by HpoT132A expression, even in stretched cells (S9E Fig). In the wing, we find that phosphomutant HpoT132A dramatically inhibits tissue growth compared with wild-type Hpo expression (S9F, S9G and S10 Figs). These results suggest that this highly conserved Akt phosphorylation site in Hpo–MST1/2 kinases (S11 Fig) may be functional in vivo, although Akt may also employ other mechanisms to regulate Hippo pathway signalling to control cell proliferation and tissue growth.

Because the Hippo pathway appears to effectively integrate PI3K–Akt signalling with either mechanical stress sensing (via Rho-kinase [Rok]–Ajuba–Wts) in the columnar cells of the wing disc or mechanical strain sensing (via reduced Hpo kinase activation) in follicle cell epithelium [89, 94], we sought to confirm that the Rok–Ajuba–Wts mechanism is indeed dispensable in follicle cells. Accordingly, mutant clones for *rok*^*2*^ or *ajuba*^*ΔII*^ induced in the follicle cell epithelium (marked by absence of red fluorescent protein [RFP]) has no effect on nuclear localisation of Yki–GFP in mechanically stretched follicle cells (S12 Fig). These results confirm that the Rok–Ajuba pathway is not required for regulation of the Hippo pathway by mechanical strain, and thus, that the Hippo pathway uses distinct mechanisms to sense stress and strain in *Drosophila*. Nevertheless, an input from PI3K–Akt is essential for Yki activation in response to either stress or strain, regardless of the mechanisms involved.

**Fig 7:**
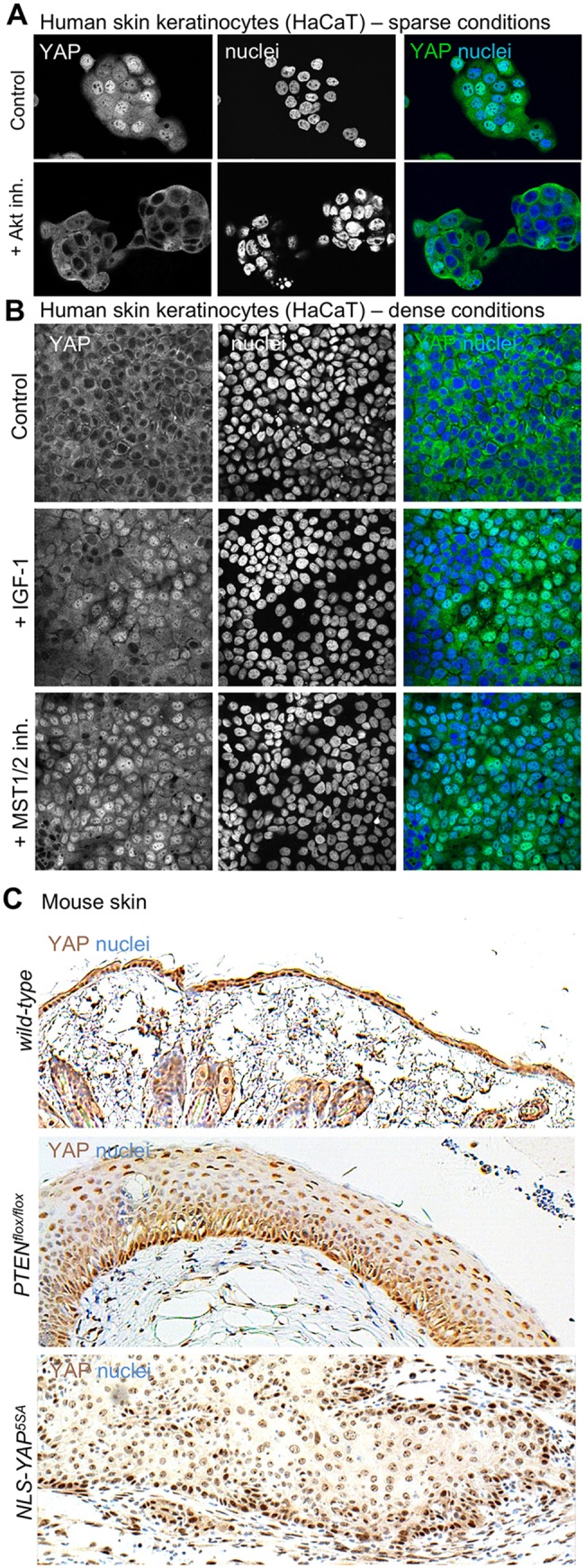
YAP integrates mechanical cues with PI3K–Akt signalling to promote cell proliferation in mammalian skin. A) Human skin keratinocytes (HaCaT) cultured in sparse conditions to induce nuclear localisation of YAP were treated with either control (DMSO) or Akt inhibitor (MK2206) at 1 μM for 4 hours prior to fixation and immunostaining for YAP, which becomes cytoplasmic upon Akt inhibition. B) Human skin keratinocytes (HaCaT) cultured in dense conditions to induce cytoplasmic localisation of YAP were treated with control (DMSO), IGF-1, or MST1/2 kinase inhibitor (XMU-MP-1) for 4 hours prior to fixation and immunostaining for YAP, which becomes nuclear upon IGF-1 activation or MST1/2 inhibition. C) Mouse skin from wild-type, skin-specific conditionally induced *PTEN* knockout or skin-specific conditionally induced nuclear *NLS–YAP–5SA*–overexpressing animals were immunostained for YAP (brown). Note similar nuclear localisation of YAP and tumour-like tissue overgrowth in both *PTEN* knockout and *NLS–YAP–5SA* skin. IGF-1, insulin-like growth factor 1; inh., inhibited; MST, Mammalian Sterile 20 kinase; *NLS*, nuclear localisation signal; PI3K, phosphatidyl inositol-3-kinase; PTEN, phosphatase and tensin homolog; YAP, Yes-associated protein.

In mammals, the skin epithelium is an example of a tissue in which both IIS via Akt [119–125] and mechanically regulated YAP and Transcriptional Activator with a PDZ domain (TAZ) [126–130] are known to promote keratinocyte cell proliferation, tissue regeneration after wounding, and tumour formation. Previous work identified a key role for PI3K and PDK1 in promoting nuclear localisation of YAP in skin keratinocytes [126] and other cell types [131]. We now find that treatment with Akt inhibitor is sufficient to prevent YAP nuclear localisation in keratinocytes, even when plated at a low density to induce mechanical stimulation via integrin adhesions (Fig 7A). We further find that treatment of densely packed keratinocytes (with cytoplasmic YAP) with IGF-1 is sufficient to promote YAP nuclear localisation in all but the most crowded cells, similar to inhibition of the Hpo orthologs MST1/2 (Fig 7B). Thus, activation of PI3K–Akt has a similar effect on YAP as inhibition of Hippo pathway signalling in these cells. Finally, in mouse skin, activation of Akt by skin-specific deletion of *PTEN* is sufficient to promote YAP nuclear localisation and tumour-like tissue overgrowth, similar to that induced by skin-specific expression of nuclear-localised YAP^5SA^ (Fig 7C). Together, these results indicate that IIS via Akt inhibits the canonical Hippo pathway to promote nuclear localisation of YAP and tissue growth in mammalian skin.

## Discussion

Our results show that the Hippo pathway has a physiological function in integrating hormonal IIS with mechanical inputs and polarity cues to control tissue growth in two different proliferating epithelia of *Drosophila* as well as in mammalian skin. Although manipulation of the Hippo pathway was previously shown to strongly affect cell proliferation and tissue growth [4–6], as well as to respond to the Fat–Dachsous planar polarity pathway [4–8], mechanical stress [89, 91–94], and mechanical strain [88, 95], it was previously unclear whether the Hippo pathway was also physiologically regulated by hormonal insulin/IGF-1 signals to control tissue growth during development. Our findings support the view that Yki/YAP normally relocalise to the nucleus to drive cell proliferation in epithelial cells subject to both mechanical stimuli and growth factor signalling via the PI3K–PDK1–Akt pathway. Below, we discuss how the Hippo pathway integrates these cues in different tissue types and different animal species. We then consider how evolutionary changes in the structure of the epidermis between *Drosophila* and mammals impact the mechanisms involved [8].

In the *Drosophila* ovarian follicle cell epithelium, our results suggest that growth of the germline stretches the surrounding follicle cells to induce nuclear localisation Yki, which activates Sd-mediated transcription to promote cell proliferation (Figs 1 and 2). Note that previous investigation with *ex*.*lacZ* failed to identify an effect of germline growth on follicle cell Yki activity, most likely because of perdurance of the *lacZ* gene product, beta-galactosidase [16]. The primary mechanism of mechanotransduction in this tissue involves the canonical Hippo pathway, which is activated by Crumbs (Crb)-mediated clustering of apical Hpo kinase containing complexes to promote Hpo dimerisation and transactivation and inactivated by mechanical strain to decluster the same complexes [88]. In addition, our results show that follicle cells must receive an insulin/IGF-1 signal that activates PI3K–PDK1–Akt to inhibit the Hpo kinase, possibly via direct phosphorylation on a conserved Akt phosphorylation motif or via other mechanisms that are still to be elucidated (Figs 3 and 4; S9–S11 Figs). In the absence of sufficient PI3K–PDK1–Akt signalling in follicle cells, the Hpo kinase becomes abnormally active, and Yki is retained in the cytoplasm even following mechanical strain (Fig 4). We note that PI3K–PDK1–Akt signalling directly affects Hpo kinase activity but that Hpo kinase activity itself promotes dimerisation because a kinase-active Hpo dimerisation sensor is much more strongly dimerised than the kinase-dead version [88]. Thus, the dimerisation and transactivation mechanism of the Hpo kinase enables it to integrate both mechanical inputs and hormonal insulin/IGF-1 signals in follicle cells. We note that the Hippo pathway continuously senses mechanical strain in the follicle cell epithelium [88] but only controls cell proliferation during the early stages (1–7) of oogenesis (Figs 1–3), after which Notch receptor and ecdysone receptor (EcR) activation terminates proliferation by inducing a switch to endoreplication [132, 133].

During *Drosophila* wing disc growth, cessation of larval feeding in the late third instar stage reduces IIS and leads to decreased Yki nuclear localisation as cell proliferation first slows and then ultimately arrests upon ecdysone-induced metamorphosis (Fig 5A). Our findings confirm previous work linking IIS via PI3K and PDK1 with regulation of the Hippo pathway in the wing [90] and extend it by providing a molecular mechanism—Akt-mediated regulation of Hpo signalling—and demonstrating the physiological significance of this mechanism in tissue growth control (Figs 5A–5D, 6A and 6B). Our results confirm that nuclear localisation of Yki occurs in both mechanically stressed cells (domains with high Rok and Myosin-II [Myo-II] along the D–V boundary) and in mechanically strained cells (strongly flattened peripodial cells), as well as cells with high Fat–Dachsous signalling (at the periphery of the wing pouch) (Figs 5A–5C, 6A and 6B; S7 Fig). Stress sensing occurs via Rok and Myo-II, which are known to promote localisation of the Wts/Large tumour suppressor (LATS) inhibitor Ajuba/LIM domain 1 (LIMD1)/Thyroid receptor interacting protein 6 (TRIP6) to adherens junctions, which promotes Yki activity in the wing disc [89, 94, 134–136] (S7B Fig). Strain sensing in peripodial cells and also follicle cells occurs via a distinct mechanism involving dilution of apical Hippo pathway components [88, 137], and thus, follicle cells do not require Rok or Ajuba to activate Yki (S12 Fig). Note that the strong Ajuba-driven nuclear localisation of Yki along the D–V boundary of the wing disc does not actually lead to pro-proliferative target gene activation because of antagonism by Notch signalling [107]. Thus, the Yki target gene *bantam*, which, along with *Myc*, mediates much of the effect on Yki on cell proliferation [87, 138, 139], is activated in a circumferential pattern at the periphery of the wing pouch and repressed medially during the early larval stages (Fig 6A)—in agreement with proposed models for mechanical stretch-induced proliferation in this tissue [110–114] and with gradients of Fat–Dachsous signalling [4–8]. Note also that Ajuba-mediated Yki activation was recently proposed to decline over time in the wing disc because of reduced mechanical stress and/or increased compression of cells [89], and our finding that increased PI3K–Akt signalling restores Yki nuclear localisation (Fig 5C and S7B Fig) suggests that integration of both mechanics and hormonal input is necessary to explain the temporal regulation of Yki.

The role of Myc as a downstream target of PI3K–Akt and Yki is particularly interesting because Myc not only stimulates cell proliferation and drives cell competition but also provides positive feedback on PI3K–Akt signalling itself [140]. Thus, nutritional regulation of growth in *Drosophila* involves insulin/IGF-1–mediated signalling via PI3K–Akt, which can stabilise the Myc protein but also drive expression of the *myc* gene via activation of Yki–Sd (in the context of sufficient mechanical input) to sustain a feed-forward loop that drives further signalling and growth. Starvation-induced loss of IIS or insufficient mechanical input due to developmental defects might thus rapidly slow growth until nutrition is located or normally coordinated development is restored.

In mammalian skin, squamous epithelial cells lack a classical apical domain typical of columnar epithelia. Instead, basal layer cells contact the underlying extracellular matrix via basal integrin adhesions, which are important for stem cell proliferation [141–143] and to promote YAP/TAZ activation via multiple mechanisms, including activation of Focal Adhesion Kinase (FAK) and Rous Sarcoma Virus Oncogene (Src)-family kinase signalling [7, 8, 93, 126]. Src can directly phosphorylate YAP on three tyrosine residues to promote its activation [144] and was also reported to inhibit the LATS1 kinase in mammals [145]. Integrin signalling can also activate PAK1 to inhibit membrane association of the key upstream Hippo pathway activator Merlin/Neurofibromatosis2 (NF2) [146–153]. Notably, integrins are generally dispensable for regulation of the Hippo pathway in *Drosophila* species, which have a columnar epidermis and can thus regulate Hippo pathway signalling via the apical domain as described above [8]. Accordingly, *Drosophila* Yki lacks the tyrosine residues phosphorylated by Src in mammalian YAP, and *Drosophila* Merlin lacks the PAK1 phosphorylation site from mammalian Merlin/NF2.

Despite this divergence in mechanotransduction mechanisms between *Drosophila* and mammals, the Akt site in Hpo is conserved in MST1/2 [115–118], and our results indicate that integration of mechanical signalling and IIS is also a fundamentally conserved feature across metazoa. As in *Drosophila*, growth-factor–induced PI3K signalling is necessary for nuclear localisation of mammalian YAP [93, 126, 131], and our data show that this effect requires Akt activity and that IGF-1 is sufficient to promote nuclear localisation of YAP in response to mechanical stimulation in human keratinocytes (Fig 7). In mice, activation of PI3K–PDK1–Akt in conditional knockout *PTEN*^*flox/flox*^ skin [154] also promotes YAP nuclear localisation and skin proliferation, similar to conditionally induced expression of activated form of YAP (nlsYAP^5SA^) (Fig 7), confirming that YAP acts as an integrator of mechanical cues and IIS via PI3K–PDK1–Akt in skin cells both in culture and in vivo.

In conclusion, a key physiological function for the Hippo pathway is to integrate input from epithelial cell polarity determinants, mechanical cues, and hormonal IIS to promote cell proliferation and tissue growth in both *Drosophila* epithelia and mammalian skin. Although mechanisms of mechanotransduction via the Hippo pathway differ in the monolayered columnar epidermis of *Drosophila* versus the stratified squamous epidermis of mammals, growth factor signalling acts via a common PI3K–PDK1–Akt-mediated mechanism to inhibit the Hippo pathway and thus promote tissue growth. Thus, the Hippo signalling pathway could be considered as one of the key effectors of Akt in tissue growth control, alongside inhibition of FOXO and the classical inhibition of TSC1/2 to activate TORC1 (S13 Fig). Notably, our results show that activation of Akt alone is not sufficient to activate Yki because some type of mechanical stimulus is also necessary. In the absence of significant mechanical inputs, Akt acts primarily via TORC1 and FOXO, and the postmitotic *Drosophila* pupal retina appears to exemplify this mode of action—a phenomenon that helped distinguish and delineate PI3K–Akt and Hippo–Yki as separate pathways in pioneering genetic screens. Nevertheless, the widespread coupling of PI3K–Akt and Hippo pathway signalling in many tissues is potentially of major significance for human cancer because activation of PI3K–Akt signalling (in *PIK3CA* or *PTEN* mutant cells) causes formation of highly proliferative malignant tumours, while mutation of *TSC1* or *TSC2* leads to formation of relatively benign tumours [75, 81]. Given the increasing evidence for a potent role of YAP/TAZ in malignant tumours, future work should examine whether the Hippo pathway might be the primary effector of PI3K–Akt signalling in human cancer and thus a critical target for therapy.

## Materials and methods

### Ethics statement

All animal-regulated procedures were carried out according to Project License constraints (PPL 70/7926) and United Kingdom Home Office guidelines and regulations. All experiments were carried out in accordance with the UK Animal Scientific Procedures Act (1986). The Francis Crick Institute Animal Ethics committee was the responsible party for approving animal care and use under this project licence. The research did not involve human participants.

### *Drosophila* genetics

Expression of UAS-driven transgenes in ovarian follicle cells was performed with the *TJ*.*Gal4* driver and in wing imaginal disc cells with the *hh*.*Gal4* driver, which is expressed in the posterior compartment, or the *nubbin*.*Gal4* (*nub*.*Gal4*) driver, which is expressed in the entire wing pouch. Depletion of Yki in the follicle cell epithelium was driven by a strong *UAS*.*Yki-RNAi* (BL34067) using the *TJ*.*Gal4* driver. Depletion of Sd in the follicle cell epithelium was driven by a strong *UAS*.*Sd-RNAi* (VDRC KK101497) using the *TJ*.*Gal4* driver. Fly crosses were kept at a temperature of 25 °C. Ovaries were dissected and immunostained from adult females 4 days after eclosion. Larvae were dissected at stage L3 of development. After dissection, samples were fixed in 4% paraformaldehyde and processed for imaging.

CRISPR/Cas9 genome editing was used to tag the C-terminus of Yki with eGFP. 100 ng/μl of guide RNA plasmid (pCFD3, Addgene 49410, encoding the gRNA 5′-TCAGGTTTGTGGGAAGACGG-3′; Addgene, Cambridge, MA, USA), plus 500 ng/μl of a homologous recombination repair template plasmid, were coinjected into nos-Cas9 embryos. The repair template contained 4.5 kb genomic DNA from the Yki locus (centred around the stop codon), with eGFP inserted in-frame after the final amino acid of Yki and before its stop codon. The gRNA target sequence in the repair template was mutagenised to prevent recutting of correctly targeted alleles. The resulting knockin allele is homozygous viable [88].

### *Drosophila* genotypes

The *Drosophila* genotypes used were as follows:

**Table:** 

Fig 1A:	*ex*^*lacZ*^*/CyO*
Fig 1B:	*Yki*:*GFP*
Fig 1C:	*w*^*iso*^
	*TJ*.*Gal4;UAS*.*Yki-RNAi BL34067/+*
	*TJ*.*Gal4;UAS*.*Sd-RNAi KK101497/+*
Fig 1D:	*w*^*iso*^
	*TJ*.*Gal4/+;UAS*.*Yki-RNAi BL34067/+*
	*TJ*.*Gal4/+;UAS*.*Sd-RNAi KK101497/+*
Fig 2A:	*Yki*:*GFP*
Fig 2B:	hsflp; *Yki*:*GFP;FRT82 akt3/FRT82 nlsRFP*
Fig 2C:	hsflp; *Yki*:*GFP; FRT82 akt3/FRT82 nlsRFP*
Fig 3A–3F:	hsflp; *Yki*:*GFP; FRT82 akt3/FRT82 nlsRFP*
Fig 4A:	*TJ*.*Gal4;UAS*.*Hippo*^*KD*^*VenusC;UAS*.*Hippo*^*KD*^*VenusN*
	*TJ*.*Gal4;UAS*.*Hippo*^*KD*^*VenusC;UAS*.*Hippo*^*KD*^*VenusN/UAS*.*DN–PI3K (p60)*
	*TJ*.*Gal4;UAS*.*HippoVenusC;UAS*.*HippoVenusN*
Fig 4B:	*TJ*.*Gal4;UAS*.*Hippo*^*KD*^*VenusC;UAS*.*Hippo*^*KD*^*VenusN/UAS*.*PTEN*
Fig 4C:	*TJ*.*Gal4;UAS*.*Hippo*^*KD*^*VenusC;UAS*.*Hippo*^*KD*^*VenusN/UAS*.*Akt–RNAi*
Fig 4D:	hsflp; *FRT82 akt3/FRT82 nlsRFP*
	hsflp; *FRT82 akt3 wtsX1/FRT82 nlsRFP*
Fig 4E:	*TJ*.*Gal4/Yki*:*GFP*;
	*TJ*.*Gal4/Yki*:*GFP;UAS*.*Hpo–RNAi/+*
	*TJ*.*Gal4/Yki*:*GFP;UAS*.*Wts–RNAi/+*
Fig 4F:	*TJ*.*Gal4/Yki*:*GFP;UAS*.*TOR*^*TED*^*/+*
	*TJ*.*Gal4/Yki*:*GFP;UAS*.*TOR–RNAi/+*
Fig 4G:	hsflp; *Yki*:*GFP;FRT82 rheb/FRT82 nlsRFP*
Fig 5A:	*Yki*:*GFP*
Fig 5B:	*ms1096*.*Gal4*/*UAS*.*akt-RNAi*;*Yki*:*GFP*
Fig 5C:	*Yki*:*GFP;hh*.*Gal4/UAS*.*InR*
	*Yki*:*GFP/UAS*.*dp110caax;hh*.*Gal4/+*
	*Yki*:*GFP/UAS*.*Akt-myr;hh*.*Gal4/+*
Fig 5D:	*hh*.*Gal4/+*
	*UAS*.*pten-RNAi;hh*.*Gal4/+*
	*UAS*.*hpo-RNAi;hh*.*Gal4/+*
	*UAS*.*akt-RNAi;hh*.*Gal4/+*
	*UAS*.*yki-RNAi;hh*.*Gal4/+*
Fig 6A:	*Sd*:*GFP*
	*Yki*:*GFP*
	*ban*.*lacZ*
Fig 6B:	*hsflp;Yki*:*GFP;FRT82 akt3/FRT82 nlsRFP*

### *Drosophila* immunohistochemistry

Ovaries and imaginal discs were dissected in PBS, fixed for 20 mins in 4% paraformaldehyde in PBS, washed for 30 minutes in PBS/0.1% Triton X-100 (PBST), and blocked for 15 minutes in 5% normal goat serum/PBST (PBST/NGS). Primary antibodies were diluted in PBST/NGS, and samples were incubated overnight at 4 °C.

The primary antibodies used were mouse anti-βgal (1:500; Promega, Madison, WI, USA), FITC-conjugated anti-GFP (1:400; Abcam, Cambridge, UK), mouse anti-Eya (1:250, DSHB), and mouse anti-Armadillo (1:100, Developmental Studies Hybrid Bank [DSHB], University of Iowa, Iowa City, IA, USA).

Secondary antibodies (all from Molecular Probes, Invitrogen, Carlsbad, CA, USA) were used at 1:500 for 2–4 hours prior to multiple washes in PBST and staining with DAPI at 1 μg/ml for 10–30 min prior to mounting on slides in Vectashield (Vector Laboratories, Burlingame, CA, USA).

### *Drosophila* image acquisition

Confocal images were taken with a Leica SP5 confocal microscope (Wetzlar, Germany) using 20× and 40× oil immersion objectives and processed with Adobe Photoshop and Fiji. Either optical cross-sections through the middle of the different tissues or the top focal plane are shown in all figures. Pictures of the adult wings mounted in Hoyer’s medium were taken on a Zeiss Axioplan 2 Imaging Microscope (Oberkochen, Germany).

### Cell culture

Human HaCAT cells (Francis Crick Institute cell services) were grown in DMEM (Gibco 41966; Gaithersburg, MD, USA) with 10% FCS and penicillin/streptomycin. All cells are subject to mycoplasma testing. Cells were treated with the Akt inhibitor MK2206 or the MST1/2 inhibitor XMU-MP-1 for 4 hours before being fixed with 4% PFA for 15 minutes before immunostaining.

### Cell culture antibodies, image acquisition, and quantification

The primary antibodies used were mouse YAP (Santa Cruz sc-101199) 1:100. Secondary antibodies were from Invitrogen and used at 1:500 for 2 hours at room temperature along with DAPI. Cell culture samples were imaged with a Leica SP5 confocal microscope using a 63× oil immersion objective and processed using Adobe Photoshop. Cells were assessed over three independent experiments, counting 200–300 cells per condition.

### Mouse experiments

*Conditional activation of Cre recombinase in adult epidermis*. Tamoxifen (20 mg/ml in con oil; Sigma-Aldrich, St. Louis, MO, USA) was injected intraperitoneally (IP) (5 μl/g body weight) for 5 consecutive days into 8- to 16-week–old control animals or transgenic animals carrying K5–CreERt Yap R26–YAP5SA–NLS to induce YAP5SA–NLS expression and analysed for LacZ (lineage tracer) expression by immunohistochemistry from 3 days thereafter [127]. Similar experiments were performed for the PTEN flox/flox conditional knockout skin, as described in [154].

### Immunohistochemistry

Mouse skin samples were harvested and fixed in neutral-buffered formaldehyde 10% vol/vol (Sigma-Aldrich) and then embedded in paraffin in a head-to-tail orientation. The tissues were processed, embedded, and sectioned at 4 μm and used for haematoxylin–eosin staining and immunohistochemistry. Sections were dewaxed in xylene and dehydrated by passage through graded alcohols to water. If required for antigen retrieval, sections were microwaved in citrate buffer (pH 6) for 15 minutes and then transferred to PBS. Endogenous peroxidase was blocked using 1.6% hydrogen peroxide in PBS for 10 minutes, followed by washing in distilled water. Species-specific blocking serum (diluted to 10% in 1% BSA) was used to block nonspecific staining in the tissue for 30 minutes. Slides were incubated with primary antibody diluted to 1:100 in 1% BSA for 1 hour at room temperature. Sections were washed in PBS prior to applying the appropriate biotinylated secondary antibody for 45 min at room temperature. Sections were then washed in PBS and then incubated in ABC (Vector Laboratories PK-6100) for 30 minutes. Following washing in PBS, DAB solution was applied for 2–5 minutes with development of the colour reaction being monitored microscopically. Slides were washed in tap water, stained with a light haematoxylin, dehydrated, cleared, and then mounted. Antibodies used for IHC were YAP Cell signalling (14074) 1/400 O/N. Images were acquired with a Zeiss light microscope using 40× and 20× objectives.

## Supporting information

S1 Fig Yki–Venus localises to the nucleus in the mechanically stretched cells of the follicle cell epithelium during *Drosophila* oogenesis. A) A Yki–Venus knockin line behaves identically to the Yki–GFP line. Note nuclear localisation in follicle cells at early stages of oogenesis. DAPI (blue) marks nuclei. B) At stage 10 of oogenesis, the Yki–Venus knockin line again behaves identically to the Yki–GFP line, showing nuclear localisation in stretch cells (anterior, left) but not columnar cells that contact the oocyte (posterior, right). GFP, green fluorescent protein; Yki, Yorkie.
(TIFF)

S2 Fig Sd localises constitutively to the nucleus in the follicle cell epithelium throughout *Drosophila* oogenesis. A) An Sd–GFP knockin line localises to the nucleus in all follicle cells at early stages of oogenesis. F-actin is costained in red. B) An Sd–GFP knockin line localises to the nucleus in all follicle cells at stages 6–10 of oogenesis. F-actin is costained in red. DAPI marks nuclei in blue. C) An Sd–GFP knockin line localises to the nucleus in all follicle cells at stage 14 of oogenesis. DAPI marks nuclei in blue. GFP, green fluorescent protein; Sd, Scalloped.
(TIFF)

S3 Fig Loss of Sd prevents Yki nuclear localisation and causes arrest of egg chamber development at stage 10. A) Expression of Sd–RNAi prevents nuclear localisation of Yki–GFP in early-stage egg chambers. Compare with Fig 1B. B) Expression of Sd–RNAi prevents nuclear localisation of Yki–GFP in late-stage egg chambers, including stretch cells at stage 10. C) Apoptosis, marked by Dcp1-positive cells, occurs in stage 10 germline cells affected by insufficiency in follicle cell numbers upon expression of Sd–RNAi. The Sd loss-of-function phenotype is a weaker version of the Yki loss-of-function phenotype; compare with Fig 1D. Dcp1, *Drosophila* Death Caspase 1; GFP, green fluorescent protein; RNAi, RNA interference; Sd, Scalloped; Yki, Yorkie.
(TIFF)

S4 Fig Tor-driven germline cell growth is required for flattening of ‘stretch cells’ at stage 9 of oogenesis at which Yki becomes strongly nuclear. A) Yki–GFP localises to the nucleus in stretch cells and to the cytoplasm in columnar cells of the follicular epithelium at stage 9 of oogenesis. DAPI marks nuclei in blue. F-actin is costained in red. B) Yki–GFP localises to the cytoplasm in all cells when germline growth is arrested by silencing of Tor by expression of *UAS*.*tor-RNAi* specifically in germline cells with the maternal *tub*.*Gal4* driver line. Note failure of stretch cells to become flattened in this stage 9 egg chamber. C) Yki–GFP localises to the cytoplasm in all cells when germline growth is arrested by silencing of Tor by expression of *UAS*.*tor-RNAi* specifically in germline cells with the maternal *tub*.*Gal4* driver line. Note failure of stretch cells to become flattened in this stage 8 egg chamber. GFP, green fluorescent protein; RNAi, RNA interference; TOR, Target of Rapamycin; *tub*.*Gal4*, *tubulin*.*Gal4*; *UAS*, Upstream activator sequence; Yki, Yorkie.
(TIFF)

S5 Fig Sd–GFP expression pattern in imaginal discs. A) Yki–GFP expression is shown in the wing disc proper, the wing disc peripodial epithelium, the eye disc, the eye disc peripodial epithelium, the leg disc, and the haltere disc. GFP, green fluorescent protein; Sd, Scalloped; Yki, Yorkie.
(TIFF)

S6 Fig Yki–GFP expression pattern in eye and leg imaginal discs. A) Eye imaginal discs at early, mid, and late third instar (L3) stages, showing frequent nuclear Yki–GFP in early stages and predominantly cytoplasmic Yki–GFP at late stages. Note the strong nuclear Yki–GFP in the flattened cells of the peripodial epithelium at the periphery. B) Leg imaginal discs at early, mid, and late third instar (L3) stages, showing frequent nuclear Yki–GFP in early stages and predominantly cytoplasmic Yki–GFP at late stages. Note the strong nuclear Yki–GFP in the flattened cells of the peripodial epithelium at the periphery. GFP, green fluorescent protein; Yki, Yorkie.
(TIFF)

S7 Fig Yki–GFP nuclear localisation reflects apical domain stretching (strain) as well as the pattern of Rok-–GFP/Myo-II–GFP/Jub accumulation (stress) at the apical domain in the wing pouch. A) Early, mid, and late third larval instar (L3) wing imaginal discs stained for Yki-–GFP knockin, endogenously expressed Myo-II/Sqh–GFP, and Rok–GFP expressed with *nub*.*Gal4*-driven *UAS*.*Rok–GFP*. Note correlation between Yki–GFP nuclear localisation and the pattern of mechanical stress and strain indicated by Myo-II/Sqh–GFP or Rok–GFP. B) Late-stage third larval instar (L3) wing imaginal disc immunostained for Jub and phospho-Src, both of which accumulate a junction in response to mechanical stress but not strain. GFP, green fluorescent protein; Jub, Ajuba; Myo-II, Myosin-II; *nub*.*Gal4*, *nubbin*.*Gal4*; Rok, Rho-kinase; Sqh, Spaghetti Squash; Src, Rous Sarcoma Virus Oncogene; Yki, Yorkie.
(TIFF)

S8 Fig Expression of a dominant-negative form of *tor* (*tor*^*TED*^) in the posterior compartment of the wing disc increases Yki nuclear translocation via feedback effects on PDK1–Akt activity. A) Upon inhibition of TOR activity by expression of *hh*.*Gal4 UAS*.*torTED*, there is an increase in Yki nuclear translocation in a group of cells. B) The *UAS*.*torTED*-driven nuclear translocation of Yki is inhibited by the PDK1 kinase inhibitor BX-795, indicating that TOR activity normally drives a negative feedback loop to inhibit PI3K–PDK1–Akt signalling, disruption of which leads to PDK1–Akt hyperactivation and Yki nuclear translocation in the wing. *hh*.*Gal4*, *hedgehog*.*Gal4*; PDK1, phosphoinositide-dependent kinase 1; PI3K, phosphatidyl inositol-3-kinase; TOR, Target of Rapamycin; *UAS*, Upstream activator sequence; Yki, Yorkie.
(TIFF)

S9 Fig An Akt phosphorylation site (T132) modulates Hpo kinase activity in vivo. A) Sequence of the Hpo kinase domain showing location of the catalytic aspartate residue adjacent to the Akt phosphorylation site. B) Conservation of the Akt phosphorylation site motif between *Drosophila* Hpo and human MST1/2, but not in the non-Hippo pathway kinases MST3/4. A pan-Akt substrate phosphospecific antibody recognises monomeric immunoprecipitated Hpo kinase but not the dimeric form, suggesting that Akt phosphorylation may inhibit Hpo dimerisation in S2 cells. C) Diagram of the Hpo kinase structure showing the surface accessibility of the Akt phosphorylation site adjacent to the ATP binding cleft. D) Close-up of the loop connecting the Akt phosphorylation site with the catalytic aspartate residue. E) Expression of wild-type Hpo from a third chromosome landing site causes a mild reduction in the number of follicle cells, with occasional gaps in the epithelium(*). Expression of phosphomutant HpoT132A from the same landing site causes a strong reduction in the number of follicle cells, with frequent gaps in the epithelium(*) and a failure of posterior cells to columnarise (arrow). Yki–GFP remains cytoplasmic, even in highly stretched cells, upon expression of HpoT132A. F) Expression of wild-type Hpo from a third chromosome landing site causes a mild reduction in wing size, while expression of phosphomutant HpoT132 from the same landing site causes a dramatic reduction in wing size. G) Quantification of F. See supplementary file S1_Data.xlsx for underlying data. GFP, green fluorescent protein; Hpo, Hippo; MST, Mammalian Sterile 20 kinase; Yki, Yorkie.
(TIFF)

S10 Fig Genetic epistasis between overexpressed active Akt and overexpressed Hpo kinases. A) Wing-specific *nub*.*Gal4*-driven expression of *UAS*.*myr–Akt* induces wing overgrowth. Overexpression of strongly active *UAS*.*hpoT132A* prevents wing growth and also prevents coexpressed *UAS*.*myr–Akt* from driving growth. B) Quantification of wing area from A. See supplementary file S1_Data.xlsx for underlying data. Hpo, Hippo; *nub*.*Gal4*, *nubbin*.*Gal4*; *UAS*, Upstream activator sequence.
(TIFF)

S11 Fig Conservation of the Akt site in MST1/2 and Hpo across metazoa. Alignment of the Akt phosphorylation consensus motif in MST1/2 but not MST3/4 orthologs across metazoans. Hpo, Hippo; MST, Mammalian Sterile 20 kinase.
(TIFF)

S12 Fig The Rok/Jub mechanical stress sensing pathway is not required for Yki–GFP nuclear localisation in stretched follicle cells. A) No effect on nuclear Yki–GFP localisation in Rok mutant (*rok*^*2*^) clones, marked by absence of nlsRFP in mechanically stretched follicle cells. B) No effect on nuclear Yki–GFP localisation in Jub mutant (*jub*^*Δ2*^) clones, marked by absence of nlsRFP in mechanically stretched follicle cells. Inset shows surface views of early stage 4 egg chamber. GFP, green fluorescent protein; Jub, Ajuba; nlsRFP, nuclear red fluorescent protein; Rok, Rho-kinase; Yki, Yorkie.
(TIFF)

S13 Fig Schematic diagram of Hippo signalling integrating PI3K-–Akt, mechanical, and polarity cues. (TIFF)

S1 Data Data underlying Figs 1A, 1B, 2A, 2C, 5A and 5B, S9G and S10B Figs. (XLSX)

## Abbreviations

4E-BP: Elongation initiation factor 4E binding protein
AEL: after egg laying
*ban*.*lacZ*: bantam.lacZ
BIFC: bimolecular fluorescence complementation
Crb: Crumbs
Dcp1: *Drosophila* Death Caspase 1
Dilp: *Drosophila* insulin-like peptide
*dp110caax*: *Drosophila* p110-caax
DREF: DNA replication element binding factor
D–V: Dorsal–Ventral
EcR: ecdysone receptor
*ex*.*lacZ*: *expanded*.*lacZ*
Eya: Eyes Absent
FAK: Focal Adhesion Kinase
FOXO: Forkhead box O
GAP: GTPase-activating protein
GFP: green fluorescent protein
*hh*.*Gal4*: *hedgehog*.*Gal4*
Hpo: Hippo
IGF-1: insulin-like growth factor 1
IIS: insulin/IGF-1 signalling
Inr: insulin/IGF-1 receptor
IP: intraperitoneally
IR: Inverted Repeat Hairpin RNAi
LATS: Large tumour suppressor
LIMD1: LIM domain 1
MST: Mammalian Sterile 20 kinase
Myo-II: Myosin-II
NF2: Neurofibromatosis2
NLS: nuclear localisation signal
nlsRFP: nuclear red fluorescent protein
*nub*.*Gal4*: *nubbin*.*Gal4*
*OvoD*: Ovo dominant
PBST: PBS/0.1% Triton X-100
PBST/NGS: normal goat serum/PBST
PDK1: phosphoinositide-dependent protein kinase 1
PH: Pleckstrin Homology
PI3K: phosphatidyl inositol-3-kinase
*PIK3CA*: PI3K catalytic subunit alpha
PIP: phosphatidyl inositol phosphate
PKB: protein kinase B
Pol: RNA Polymerase
PRAS40: 40-kDa proline-rich Akt substrate
PTEN: phosphatase and tensin homolog
RFP: red fluorescent protein
Rheb: Ras-homolog enriched in the brain
RNAi: RNA interference
Rok: Rho-kinase
S6K: Ribosomal protein S6 kinase
Sd: Scalloped
Src: Rous Sarcoma Virus Oncogene
TAZ: Transcriptional Activator with a PDZ domain
TEAD: TEA domain
TIF: Transcription intermediary factor
*tj*.*Gal4*: *trafficjam*.*Gal4*
TOR: Target of Rapamycin
TORC1: TOR complex 1
TRIP6: Thyroid receptor interacting protein 6
TSC: Tuberous Sclerosis Complex
*UAS*: upstream activator sequence
UBF: Upstream Binding Factor
Wts: Warts
WWTR1: WW-domain–containing transcriptional coactivator
YAP: Yes-associated protein
Yki: Yorkie
