# Supplementary figures and images for: The Hippo pathway integrates PI3K–Akt signals with mechanical and polarity cues to control tissue growth
Source: PLoS Biol. 2019 Oct 15;17(10):e3000509. doi: 10.1371/journal.pbio.3000509 (PMC6814241; doi:10.1371/journal.pbio.3000509)

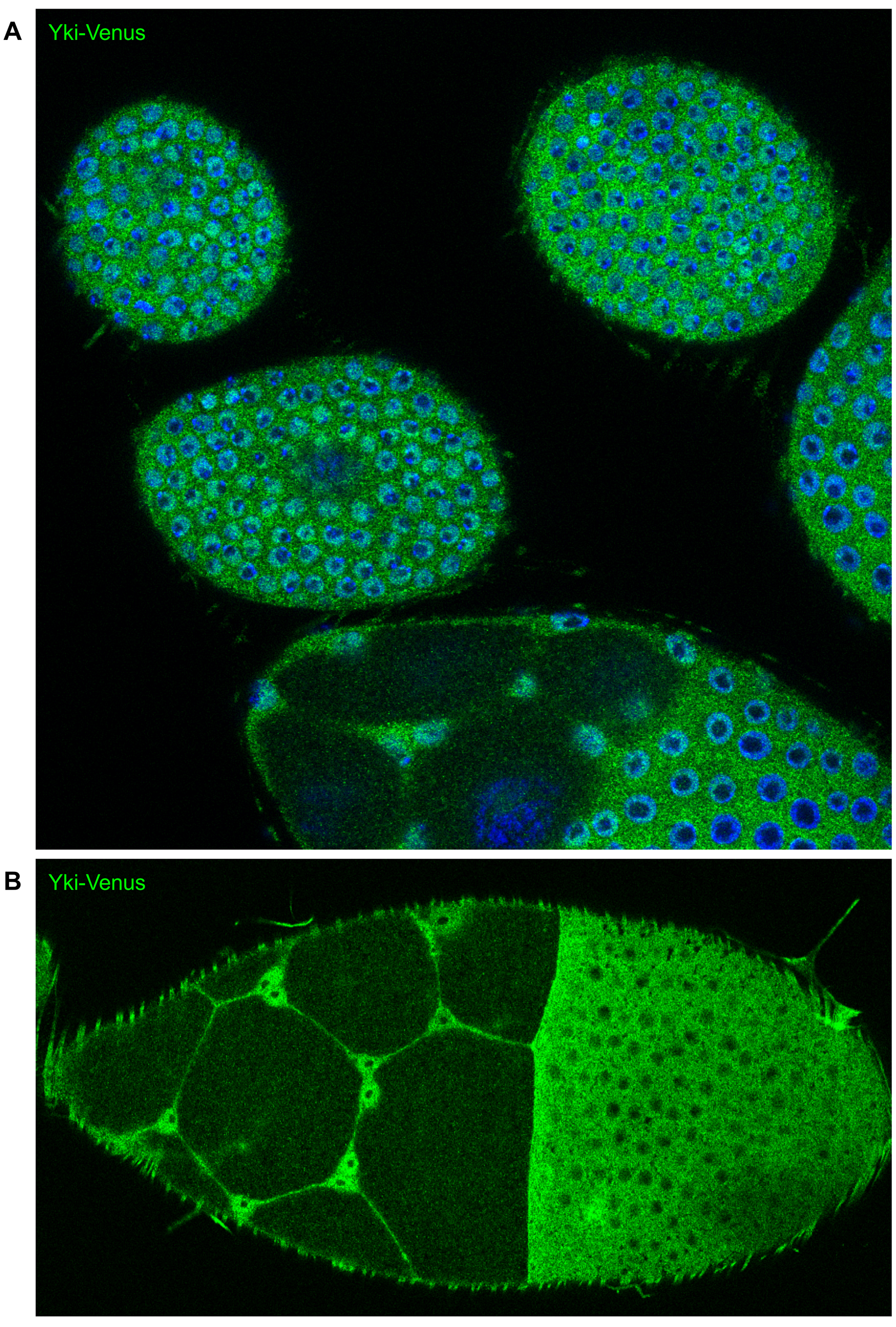

Supplement: S1 Fig — A) A Yki–Venus knockin line behaves identically to the Yki–GFP line. Note nuclear localisation in follicle cells at early stages of oogenesis. DAPI (blue) marks nuclei. B) At stage 10 of oogenesis, the Yki–Venus knockin line again behaves identically to the Yki–GFP line, showing nuclear localisation in stretch cells (anterior, left) but not columnar cells that contact the oocyte (posterior, right). GFP, green fluorescent protein; Yki, Yorkie. (TIFF) [file pbio.3000509.s001.tiff]

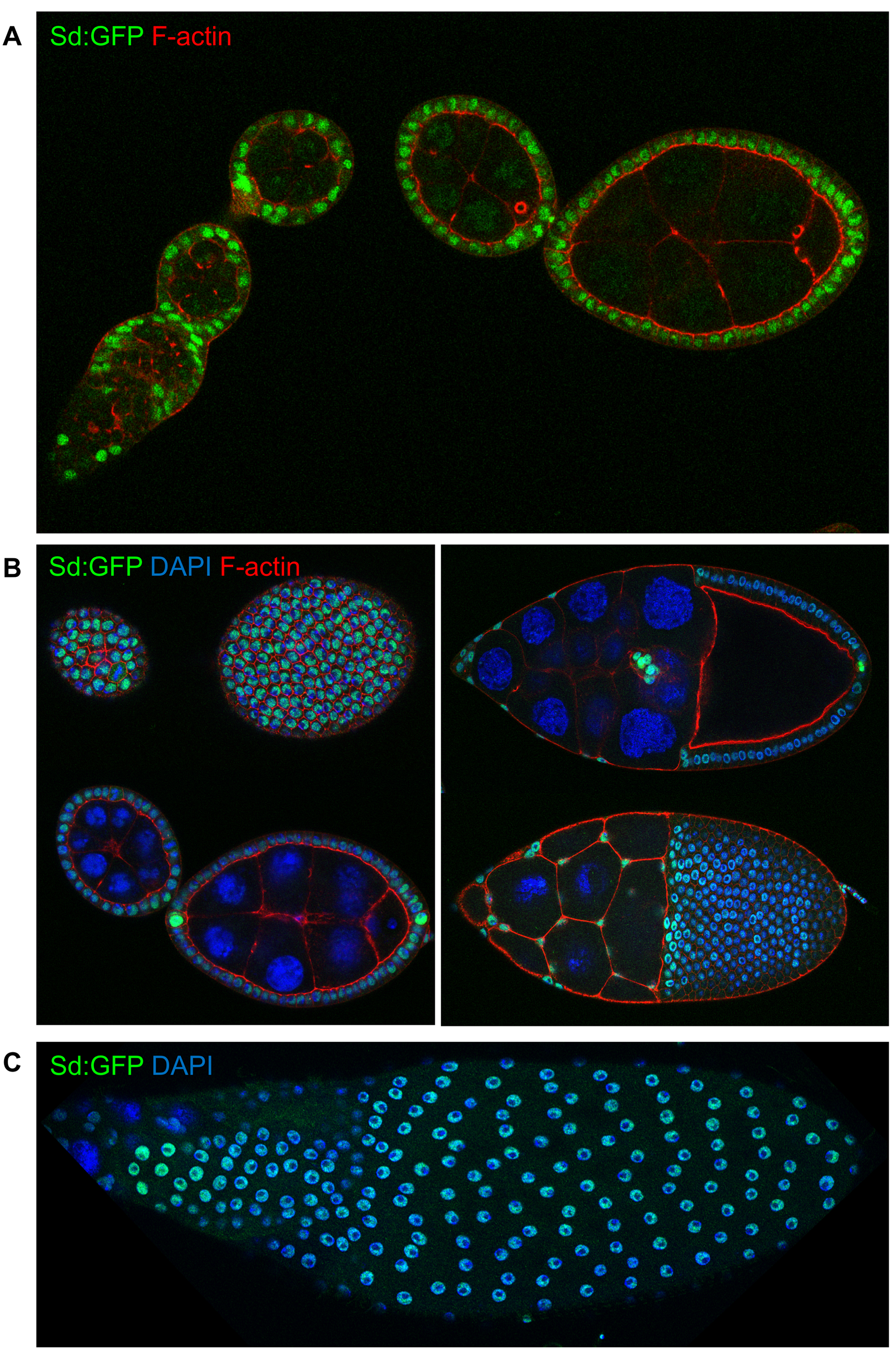

Supplement: S2 Fig — A) An Sd–GFP knockin line localises to the nucleus in all follicle cells at early stages of oogenesis. F-actin is costained in red. B) An Sd–GFP knockin line localises to the nucleus in all follicle cells at stages 6–10 of oogenesis. F-actin is costained in red. DAPI marks nuclei in blue. C) An Sd–GFP knockin line localises to the nucleus in all follicle cells at stage 14 of oogenesis. DAPI marks nuclei in blue. GFP, green fluorescent protein; Sd, Scalloped. (TIFF) [file pbio.3000509.s002.tiff]

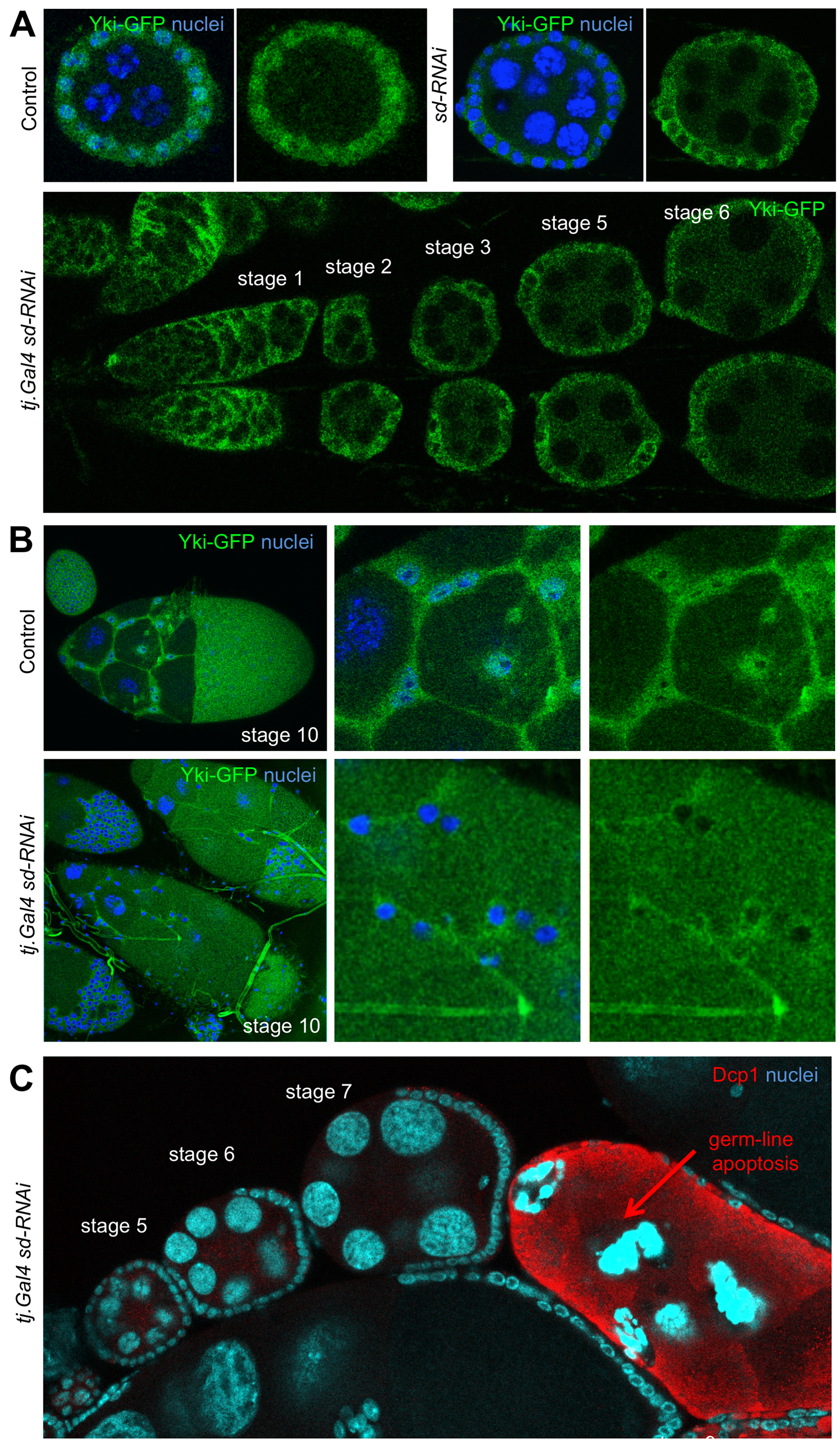

Supplement: S3 Fig — A) Expression of Sd–RNAi prevents nuclear localisation of Yki–GFP in early-stage egg chambers. Compare with Fig 1B. B) Expression of Sd–RNAi prevents nuclear localisation of Yki–GFP in late-stage egg chambers, including stretch cells at stage 10. C) Apoptosis, marked by Dcp1-positive cells, occurs in stage 10 germline cells affected by insufficiency in follicle cell numbers upon expression of Sd–RNAi. The Sd loss-of-function phenotype is a weaker version of the Yki loss-of-function phenotype; compare with Fig 1D. Dcp1, Drosophila Death Caspase 1; GFP, green fluorescent protein; RNAi, RNA interference; Sd, Scalloped; Yki, Yorkie. (TIFF) [file pbio.3000509.s003.tiff]

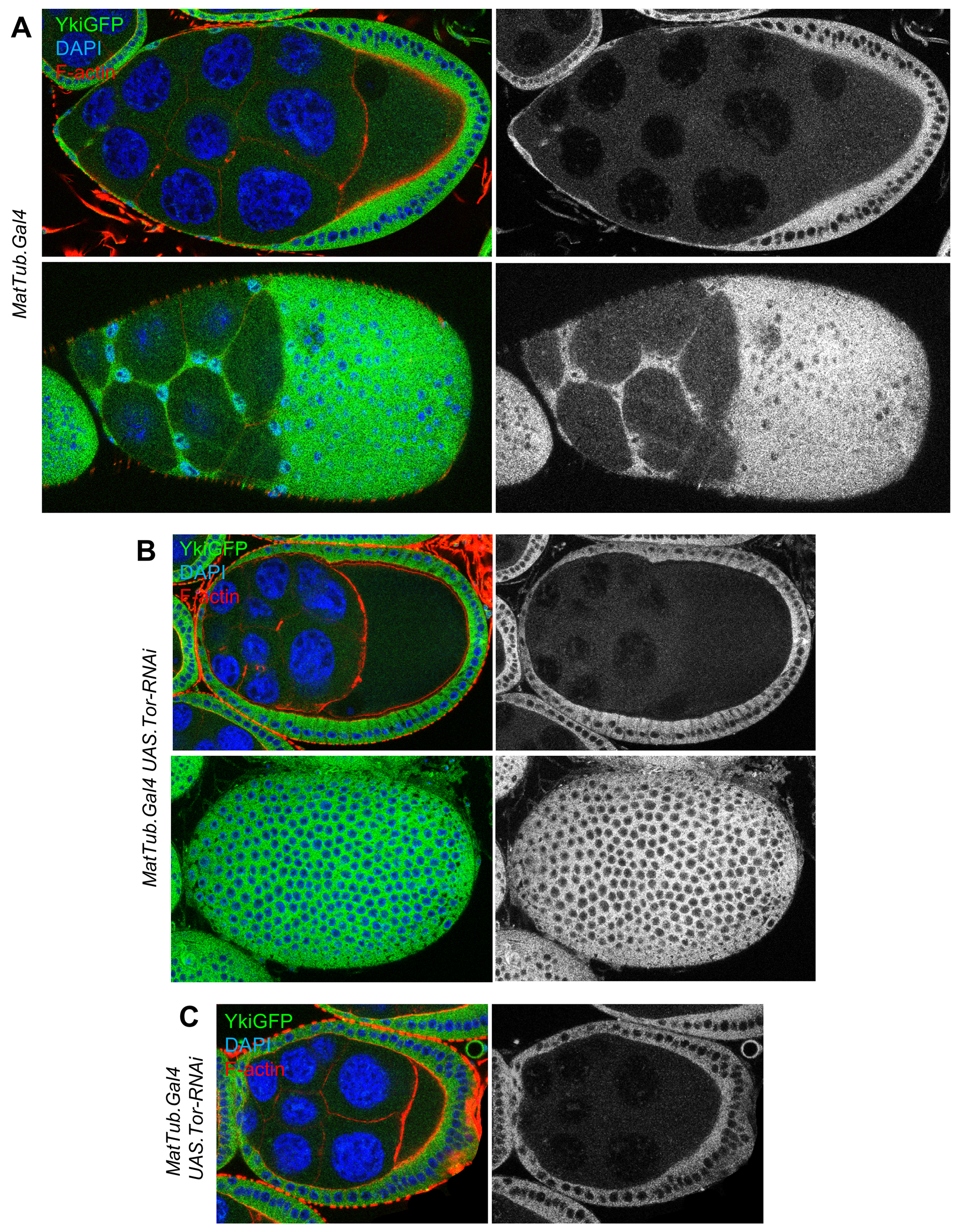

Supplement: S4 Fig — A) Yki–GFP localises to the nucleus in stretch cells and to the cytoplasm in columnar cells of the follicular epithelium at stage 9 of oogenesis. DAPI marks nuclei in blue. F-actin is costained in red. B) Yki–GFP localises to the cytoplasm in all cells when germline growth is arrested by silencing of Tor by expression of UAS.tor-RNAi specifically in germline cells with the maternal tub.Gal4 driver line. Note failure of stretch cells to become flattened in this stage 9 egg chamber. C) Yki–GFP localises to the cytoplasm in all cells when germline growth is arrested by silencing of Tor by expression of UAS.tor-RNAi specifically in germline cells with the maternal tub.Gal4 driver line. Note failure of stretch cells to become flattened in this stage 8 egg chamber. GFP, green fluorescent protein; RNAi, RNA interference; TOR, Target of Rapamycin; tub.Gal4, tubulin.Gal4; UAS, Upstream activator sequence; Yki, Yorkie. (TIFF) [file pbio.3000509.s004.tiff]

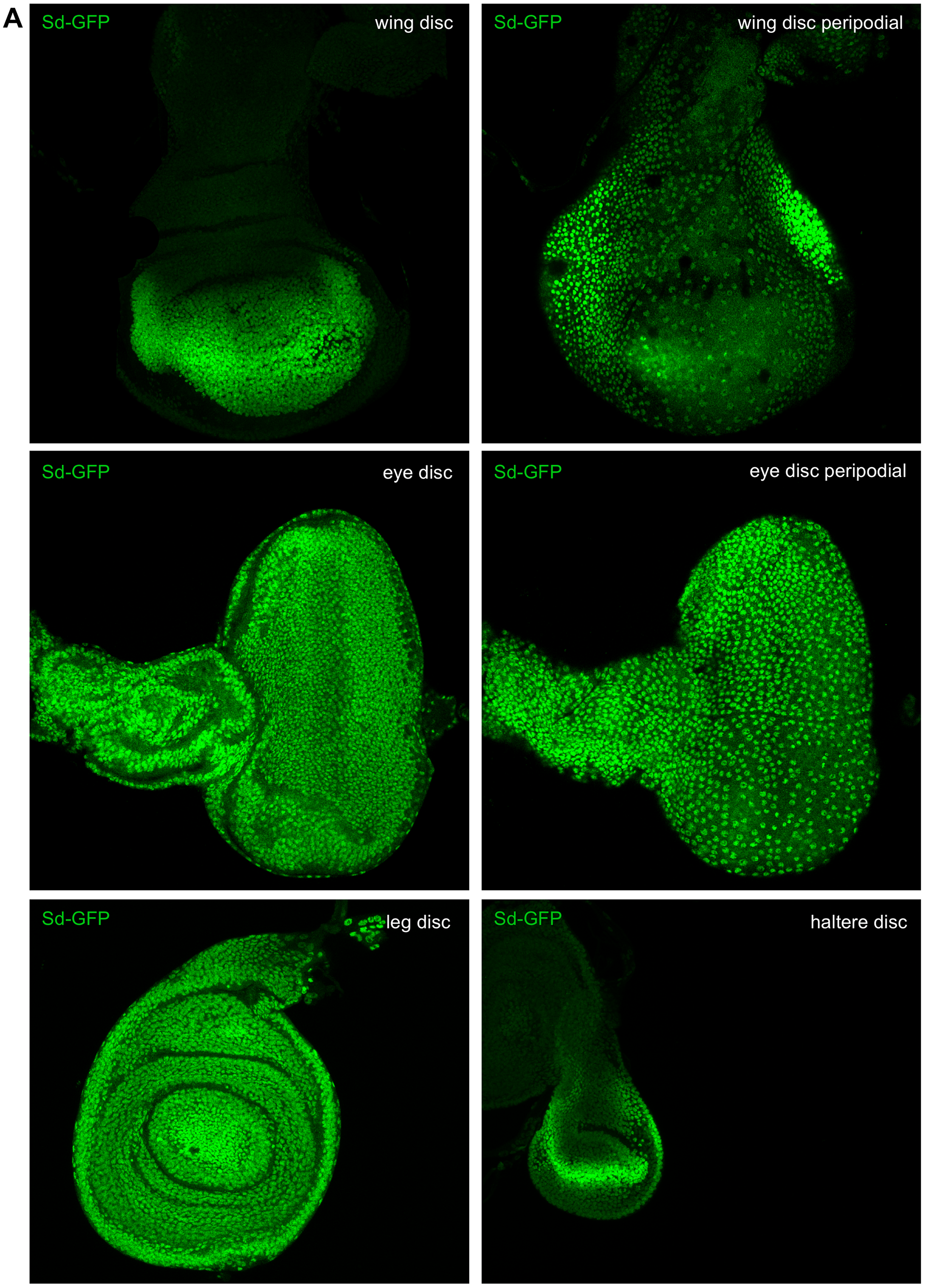

Supplement: S5 Fig — A) Yki–GFP expression is shown in the wing disc proper, the wing disc peripodial epithelium, the eye disc, the eye disc peripodial epithelium, the leg disc, and the haltere disc. GFP, green fluorescent protein; Sd, Scalloped; Yki, Yorkie. (TIFF) [file pbio.3000509.s005.tiff]

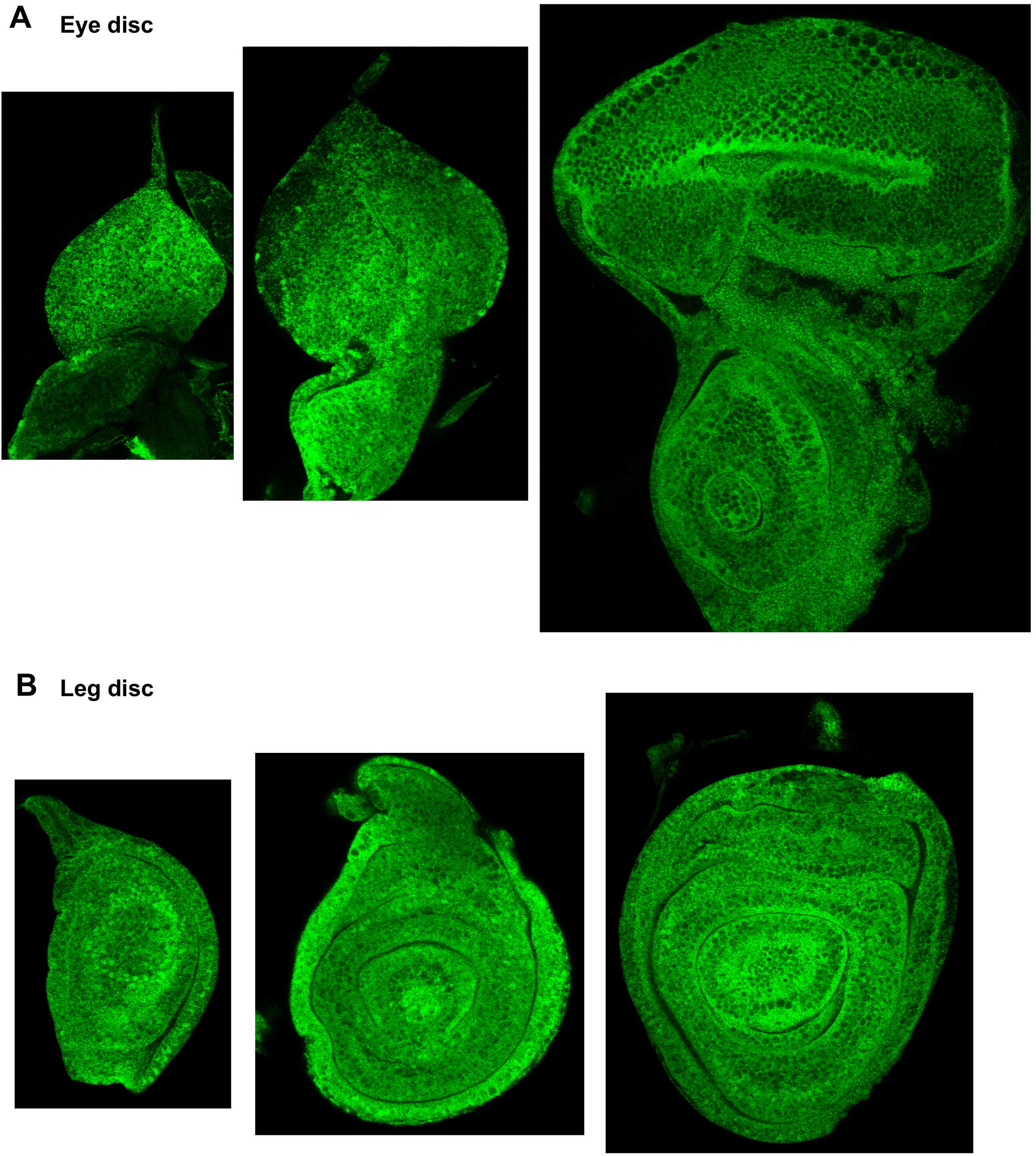

Supplement: S6 Fig — A) Eye imaginal discs at early, mid, and late third instar (L3) stages, showing frequent nuclear Yki–GFP in early stages and predominantly cytoplasmic Yki–GFP at late stages. Note the strong nuclear Yki–GFP in the flattened cells of the peripodial epithelium at the periphery. B) Leg imaginal discs at early, mid, and late third instar (L3) stages, showing frequent nuclear Yki–GFP in early stages and predominantly cytoplasmic Yki–GFP at late stages. Note the strong nuclear Yki–GFP in the flattened cells of the peripodial epithelium at the periphery. GFP, green fluorescent protein; Yki, Yorkie. (TIFF) [file pbio.3000509.s006.tiff]

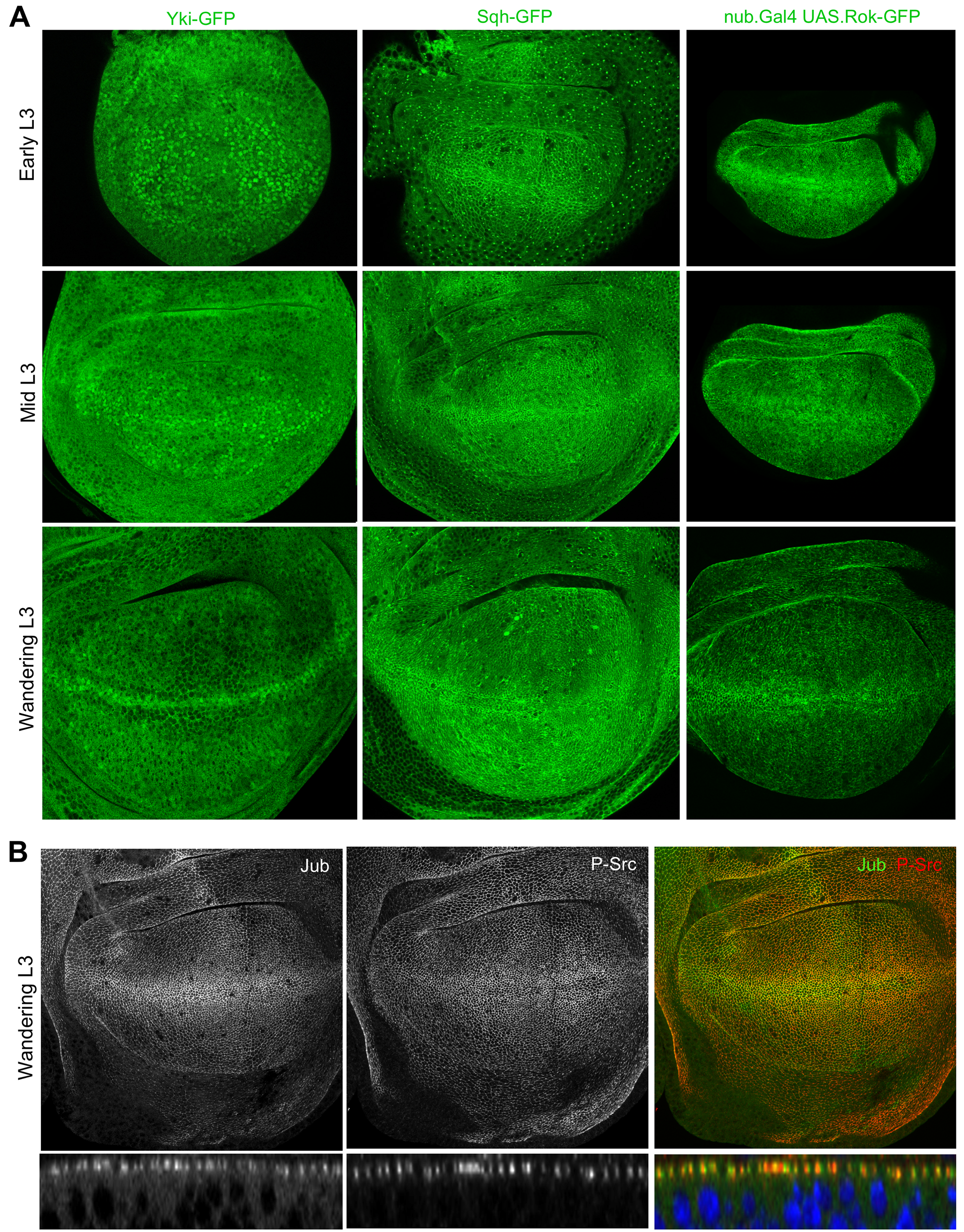

Supplement: S7 Fig — A) Early, mid, and late third larval instar (L3) wing imaginal discs stained for Yki-–GFP knockin, endogenously expressed Myo-II/Sqh–GFP, and Rok–GFP expressed with nub.Gal4-driven UAS.Rok–GFP. Note correlation between Yki–GFP nuclear localisation and the pattern of mechanical stress and strain indicated by Myo-II/Sqh–GFP or Rok–GFP. B) Late-stage third larval instar (L3) wing imaginal disc immunostained for Jub and phospho-Src, both of which accumulate a junction in response to mechanical stress but not strain. GFP, green fluorescent protein; Jub, Ajuba; Myo-II, Myosin-II; nub.Gal4, nubbin.Gal4; Rok, Rho-kinase; Sqh, Spaghetti Squash; Src, Rous Sarcoma Virus Oncogene; Yki, Yorkie. (TIFF) [file pbio.3000509.s007.tiff]

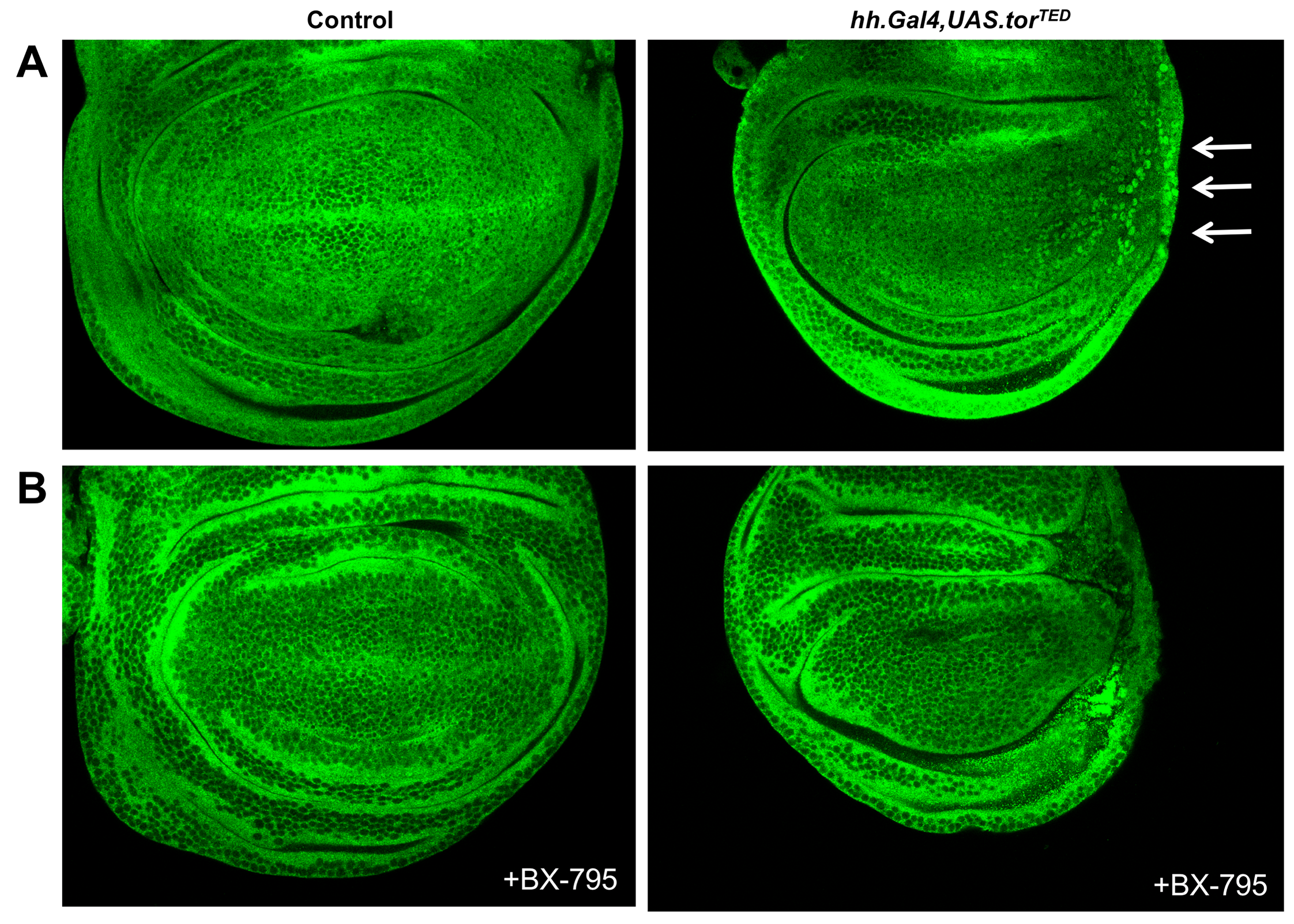

Supplement: S8 Fig — A) Upon inhibition of TOR activity by expression of hh.Gal4 UAS.torTED, there is an increase in Yki nuclear translocation in a group of cells. B) The UAS.torTED-driven nuclear translocation of Yki is inhibited by the PDK1 kinase inhibitor BX-795, indicating that TOR activity normally drives a negative feedback loop to inhibit PI3K–PDK1–Akt signalling, disruption of which leads to PDK1–Akt hyperactivation and Yki nuclear translocation in the wing. hh.Gal4, hedgehog.Gal4; PDK1, phosphoinositide-dependent kinase 1; PI3K, phosphatidyl inositol-3-kinase; TOR, Target of Rapamycin; UAS, Upstream activator sequence; Yki, Yorkie. (TIFF) [file pbio.3000509.s008.tiff]

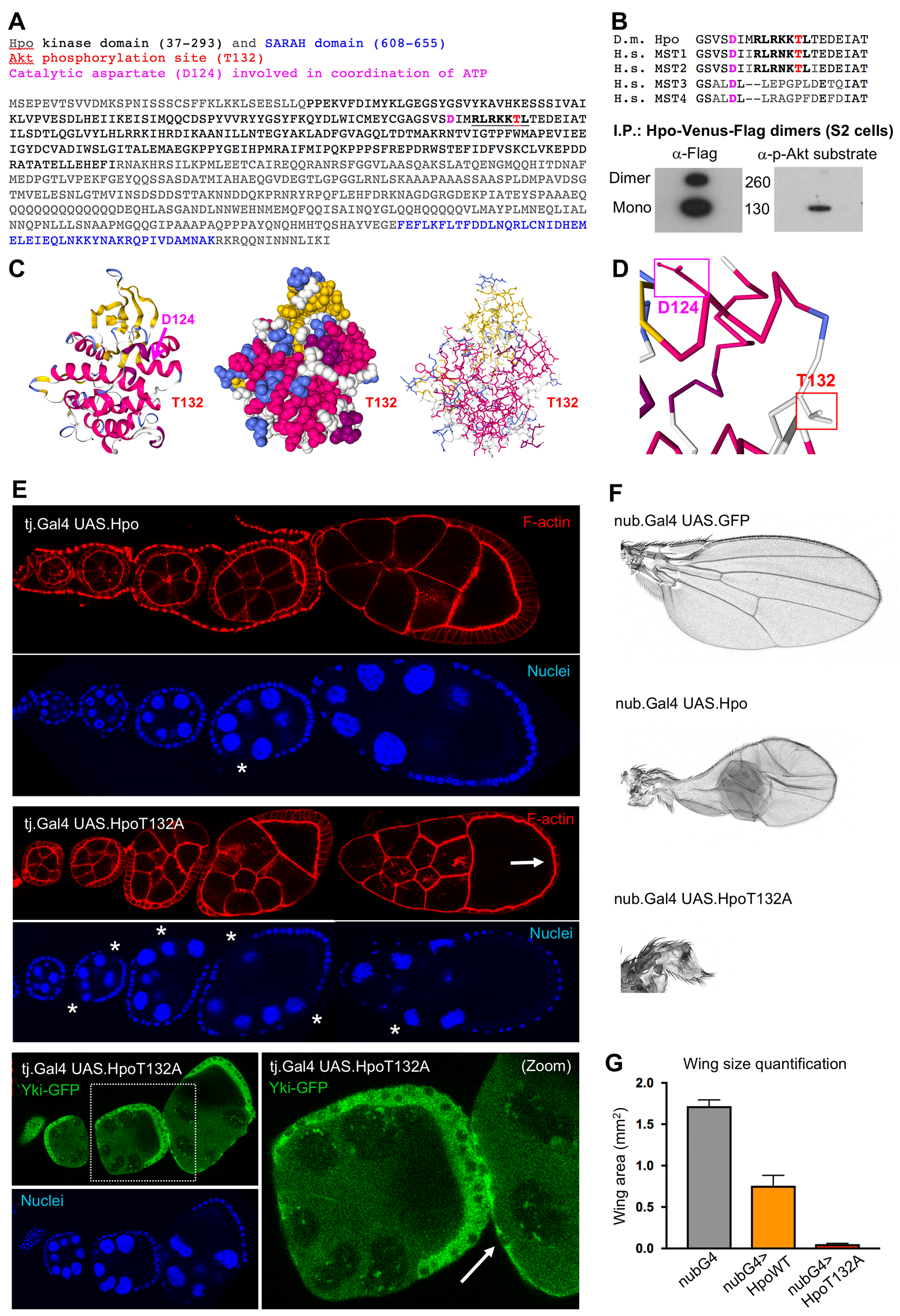

Supplement: S9 Fig — A) Sequence of the Hpo kinase domain showing location of the catalytic aspartate residue adjacent to the Akt phosphorylation site. B) Conservation of the Akt phosphorylation site motif between Drosophila Hpo and human MST1/2, but not in the non-Hippo pathway kinases MST3/4. A pan-Akt substrate phosphospecific antibody recognises monomeric immunoprecipitated Hpo kinase but not the dimeric form, suggesting that Akt phosphorylation may inhibit Hpo dimerisation in S2 cells. C) Diagram of the Hpo kinase structure showing the surface accessibility of the Akt phosphorylation site adjacent to the ATP binding cleft. D) Close-up of the loop connecting the Akt phosphorylation site with the catalytic aspartate residue. E) Expression of wild-type Hpo from a third chromosome landing site causes a mild reduction in the number of follicle cells, with occasional gaps in the epithelium(*). Expression of phosphomutant HpoT132A from the same landing site causes a strong reduction in the number of follicle cells, with frequent gaps in the epithelium(*) and a failure of posterior cells to columnarise (arrow). Yki–GFP remains cytoplasmic, even in highly stretched cells, upon expression of HpoT132A. F) Expression of wild-type Hpo from a third chromosome landing site causes a mild reduction in wing size, while expression of phosphomutant HpoT132 from the same landing site causes a dramatic reduction in wing size. G) Quantification of F. See supplementary file S1_Data.xlsx for underlying data. GFP, green fluorescent protein; Hpo, Hippo; MST, Mammalian Sterile 20 kinase; Yki, Yorkie. (TIFF) [file pbio.3000509.s009.tiff]

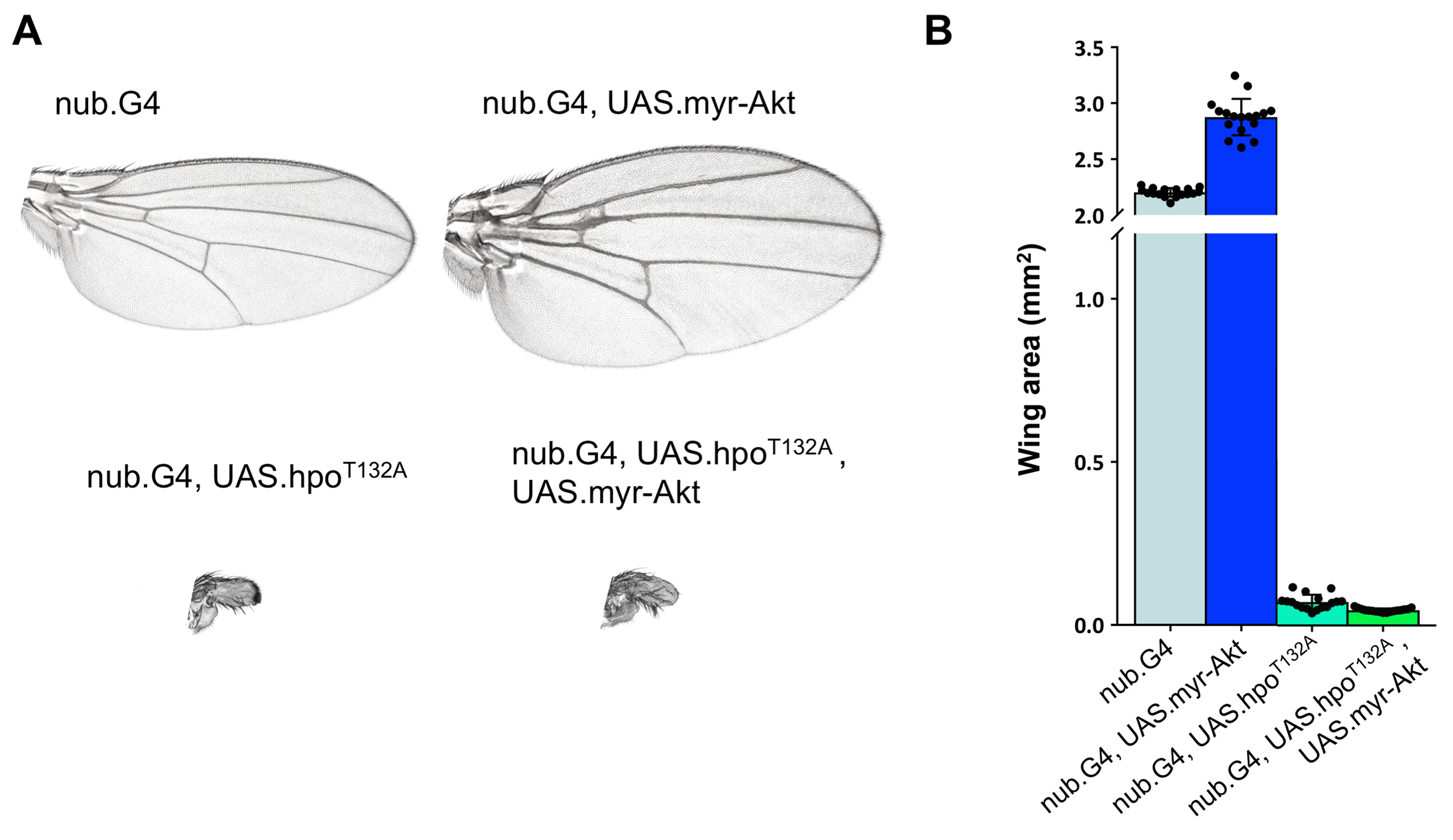

Supplement: S10 Fig — A) Wing-specific nub.Gal4-driven expression of UAS.myr–Akt induces wing overgrowth. Overexpression of strongly active UAS.hpoT132A prevents wing growth and also prevents coexpressed UAS.myr–Akt from driving growth. B) Quantification of wing area from A. See supplementary file S1_Data.xlsx for underlying data. Hpo, Hippo; nub.Gal4, nubbin.Gal4; UAS, Upstream activator sequence. (TIFF) [file pbio.3000509.s010.tiff]

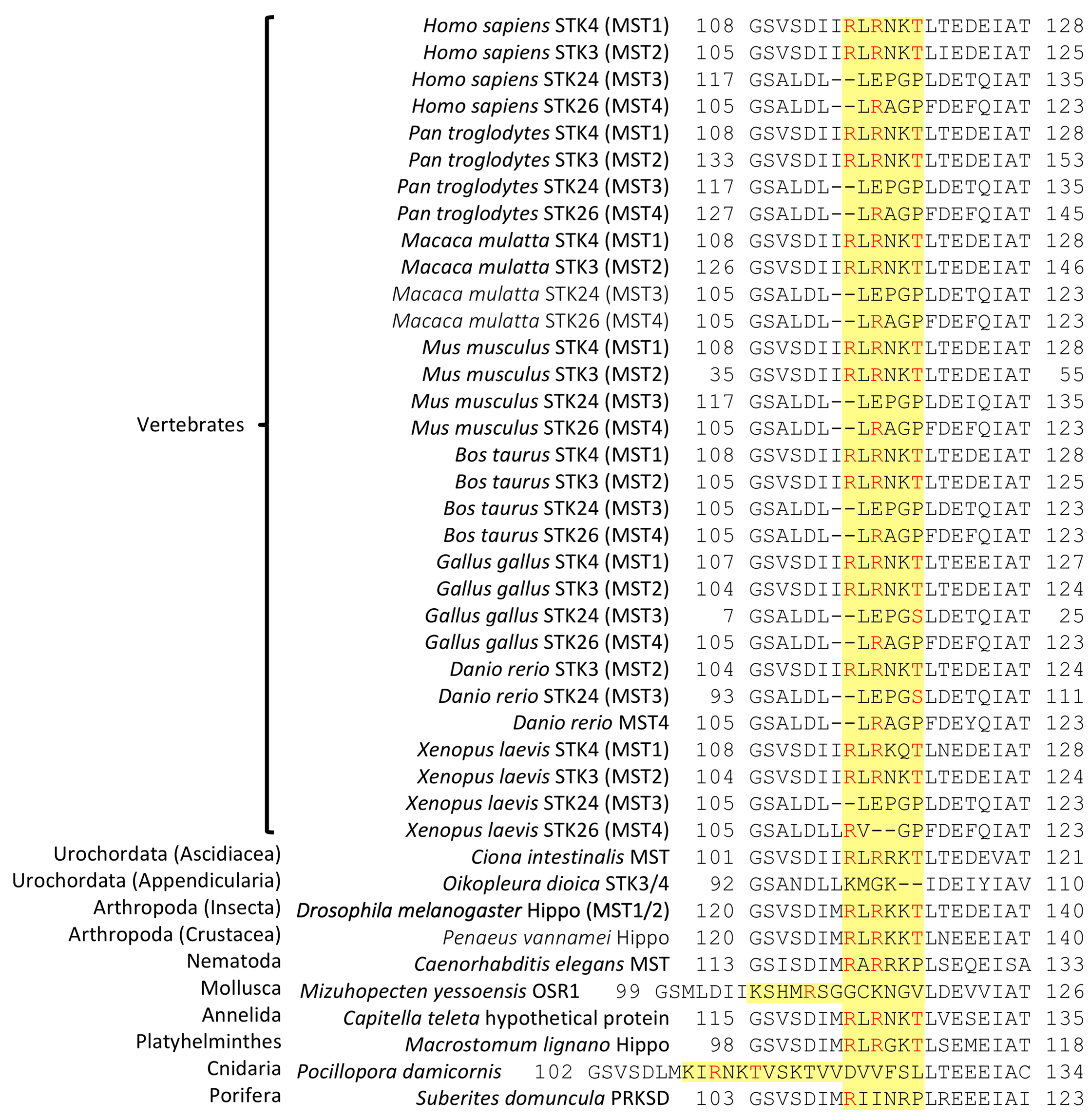

Supplement: S11 Fig — Alignment of the Akt phosphorylation consensus motif in MST1/2 but not MST3/4 orthologs across metazoans. Hpo, Hippo; MST, Mammalian Sterile 20 kinase. (TIFF) [file pbio.3000509.s011.tiff]

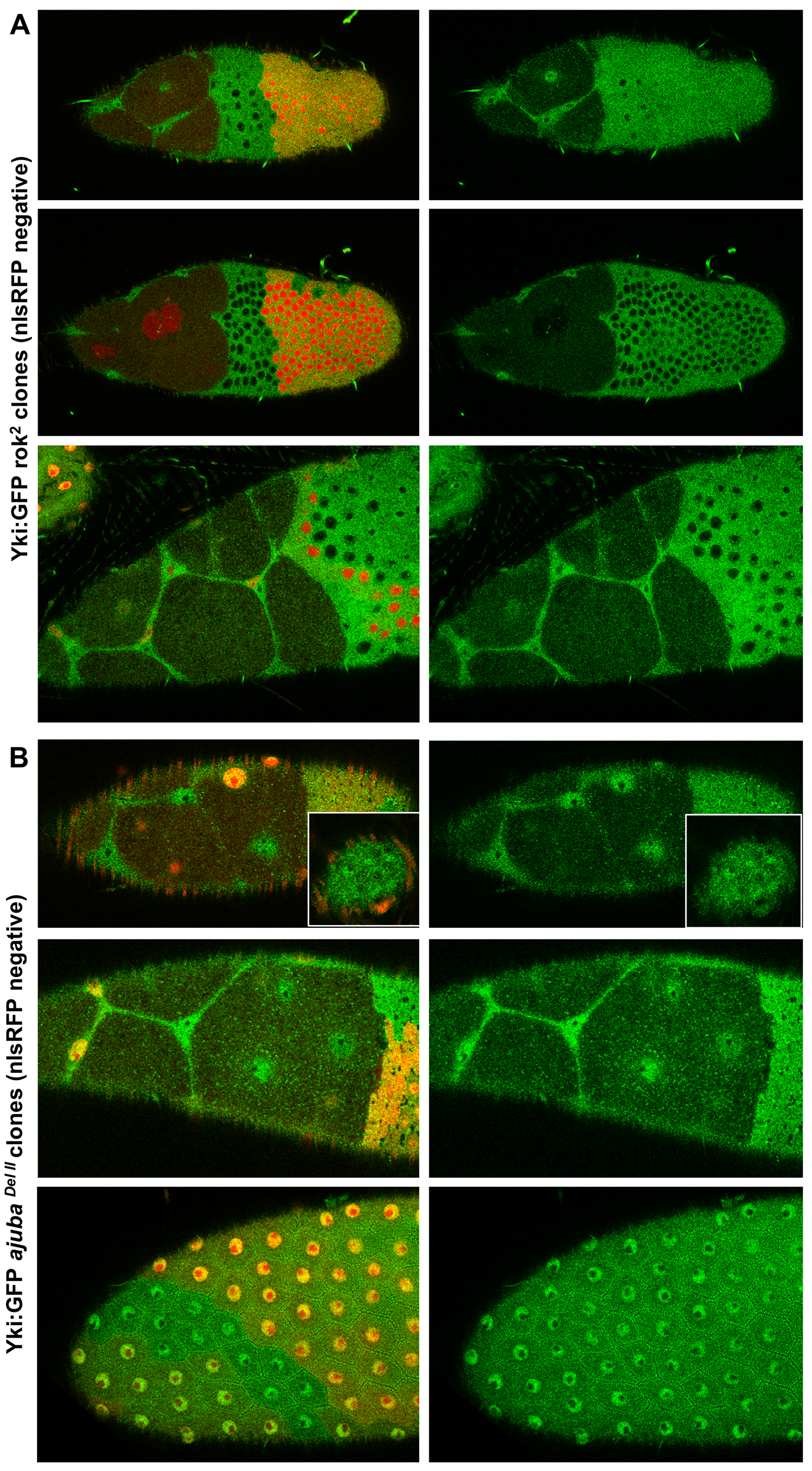

Supplement: S12 Fig — A) No effect on nuclear Yki–GFP localisation in Rok mutant (rok2) clones, marked by absence of nlsRFP in mechanically stretched follicle cells. B) No effect on nuclear Yki–GFP localisation in Jub mutant (jubΔ2) clones, marked by absence of nlsRFP in mechanically stretched follicle cells. Inset shows surface views of early stage 4 egg chamber. GFP, green fluorescent protein; Jub, Ajuba; nlsRFP, nuclear red fluorescent protein; Rok, Rho-kinase; Yki, Yorkie. (TIFF) [file pbio.3000509.s012.tiff]

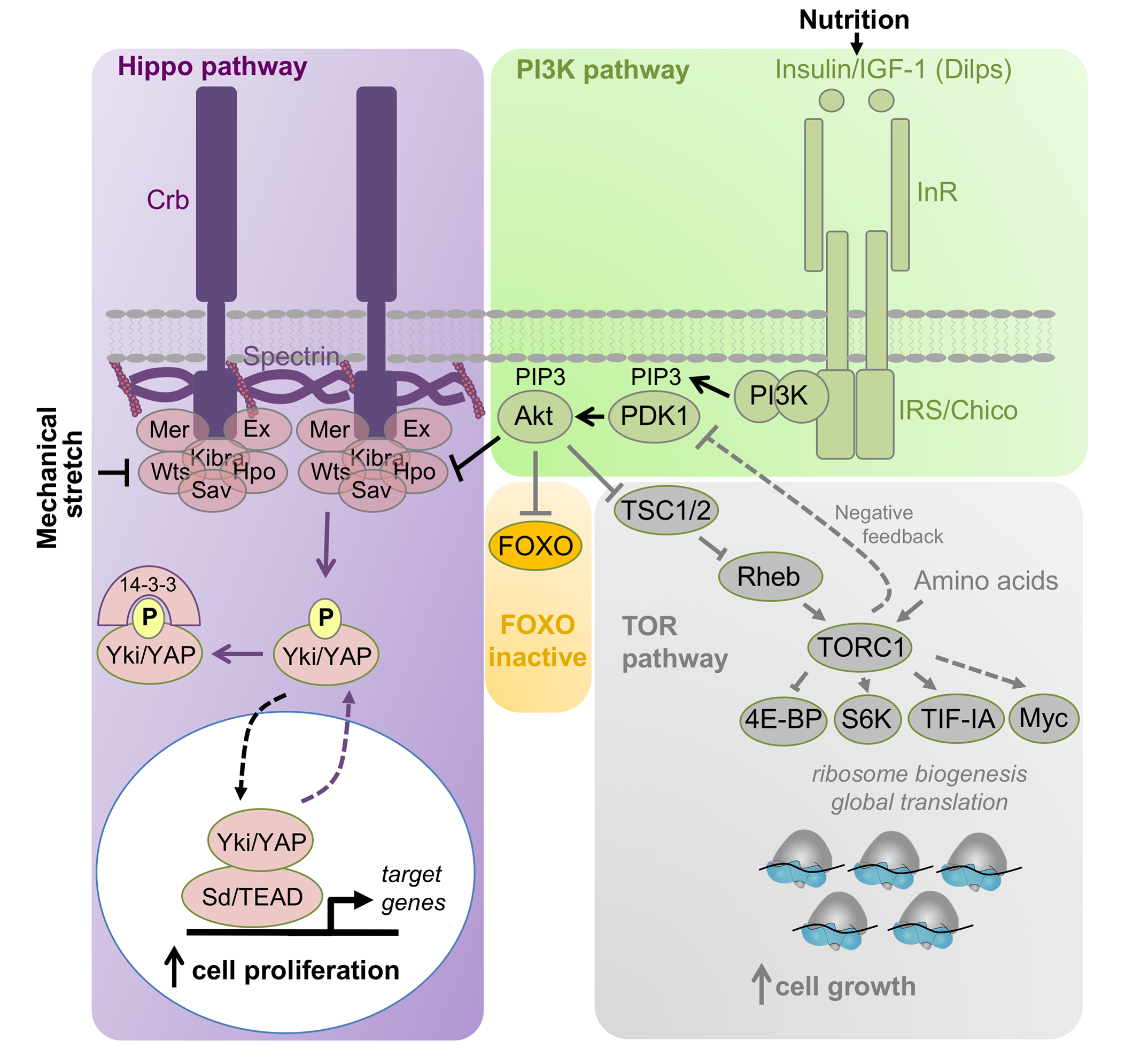

Supplement: S13 Fig — (TIFF) [file pbio.3000509.s013.tiff]
